# Investigations on the Recovery of Different Structure Types of Polyoxometalates from Aqueous Solution Using Nanofiltration Membranes

**DOI:** 10.1021/acsomega.6c00086

**Published:** 2026-04-04

**Authors:** Leon Schidowski, Luca F. Friedrich, Andreas H. Pawlig, Dorothea Voß, Jakob Albert

**Affiliations:** Institute of Technical and Macromolecular Chemistry, University of Hamburg, 20146 Hamburg, Germany

## Abstract

Polyoxometalates (POMs) are promising compounds for green and sustainable chemistry, particularly for the valorization of biomass. When used in aqueous solutions for various bioprocesses, the main challenge lies in the efficient and cost-effective recovery and separation of the POM from the heterogeneous reaction mixture. A solution could be the use of nanofiltration membranes, which were applied for different POM structure types in this work. Herein, the use of different commercial polymer membranes for the recovery of various POM structure types from an aqueous solution was investigated. Using the optimal TS-80 membrane, various parameters like concentration, flow rate, and pressure, as well as membrane stability, were analyzed to gain a fundamental understanding of POM recovery via nanofiltration. Additionally, the POM structures before and after the membrane separation process were analyzed to confirm their integrity. Through the use of nanofiltration, an efficient purification method for processing POM-based aqueous solutions could be implemented.

## Graphical abstract

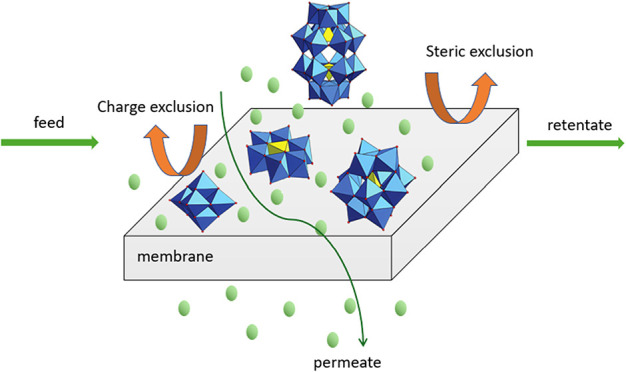

## 1 Introduction

Polyoxometalates (POMs) are a well-known class of inorganic compounds with a variety of applications in analytical chemistry, catalysis, biology, and even medicine.^1–4^ Their stability, particularly in aqueous media, and their molecular properties make them very useful for various biogenic transformations. Processes can easily be based on POMs since their acidity, the reduction potential, and other properties can be controlled by design.^5,6^ Especially in the field of biomass valorization, POMs show great potential. For example, POMs can be used for the aerobic oxidation of renewable furfural to maleic acid or to oxidatively degrade Kraft lignin in methanolic solution to monoaromatics like methyl vanillate.^7,8^ The *OxFA* process, wherein various biobased substrates can be converted into formic acid, has already been commercialized.^9–12^ Heterogenization strategies have been employed to recover POMs immobilized on various supports, such as functionalized polymers, carbon nanotubes, TiO_2_, or encapsulated POMs.^13–18^ However, leaching of the POM due to weak interactions between the POM and the support material still occurs, making regeneration of the POM challenging. A common method for the recovery in solution is liquid–liquid extraction, which is feasible but often involves the costly purification of extraction agents or the use of volatile organic compounds, which are often detrimental to the environment.^19,20^ Distillation, crystallization, and chromatography are very energy-intensive methods, and the latter is not scalable. Membrane techniques are a well-known established method for purifying mainly aqueous phases, and are attracting increasing interest in the field of homogeneous catalysis due to their low energy cost and easy scalability.^21^ For instance, separation and purification of glycerol using membranes is already an established process in biodiesel production.^22^

In terms of POMs, very little is known about applying membranes for their recovery. Pioneering this field is the group around Kahloul et al., who used Keggin POM-assisted ultrafiltration to remove dyes from aqueous solutions, achieving a rejection of >90% for lacunary and Keggin-type POM/dye complexes.^23^ The group of Albert et al. has applied this technique to POM recovery from the extractive-catalytic oxidative desulfurization (ECODS) of fuels,^24,25^ as well as using the membrane process not only to recover the Keggin-type POM catalyst from reaction solutions, but also to purify the POMs after synthesis.^26^ Since the synthesis procedure requires the adjustment of a defined pH value, alkali salts are present in addition to the POM. After four diafiltration cycles, the sodium concentration could be reduced below 0.5 g·L^–1^ with an initial concentration of 17.6 g·L^–1^ using the DK-series membrane.^26^ In several recyclability studies, the Keggin-type POM H_4_PVMo_11_O_40_ could be recycled over three times for the conversion of glucose-based humins to carboxylic esters.^27^ The XN-45 membrane used for nanofiltration was able to reject >99% of the POM and therefore to separate the products in the permeate. Additionally, the membrane performance remained stable for 166 h time on stream.

Since so far membrane purification was only used for Keggin-type POMs, the same method was applied to other POM structure types, such as Lindqvist, Anderson-Evans, and Wells–Dawson, in this study. By comparing different structure types that differ in size, charge, and geometry, this study should provide further insight into the POM recovery mechanism via nanofiltration. To the best of our knowledge, this is the first time that such a rigorous investigation of various POM structure types has been carried out. All of these structures demonstrate promising potential as catalysts in biomass conversion processes, especially when some of the framework metals are substituted by V.^28–34^ For example, a V-substituted Lindqvist-type POM (K_5_V_3_W_3_O_19_) has been successfully applied for the fractionated catalytic oxidation of lignocellulosic biomass to formic acid and cellulose,^28,29^ whereas a V-substituted Lindqvist-type Polyoxotungstate [VW_5_O_19_]^3–^ has been used for cyclooctene epoxidation by H_2_O_2_.^30^ Moreover, the Wells–Dawson-type POM (Na_9_P_2_V_3_W_15_O_62_) has been used for the selective catalytic oxidation of glucose to acetic acid^31^ or the conversion of lignin to form aromatic monomers with yields up to 24%.^32^ V-substituted Anderson-Evans-type molybdo- and tungstotellurates can catalyze the oxidation of furan derivatives to formic and maleic acid^33^ or enable efficient oxidative cleavage of β-O-4 linkages in lignin model compounds.^34^

The aim of the present study is to gain a fundamental understanding of the mechanisms leading to a complete recovery of POMs by nanofiltration and to investigate whether the recovery differs depending on the respective POM structure type.

## 2 Experimental Section

### 2.1 Chemicals

All chemicals were purchased commercially and used without further purification. Sodium tungstate dihydrate (99%) and acetic acid (99%) were sourced from *VWR Chemicals*. Sodium metavanadate and vanadium pentoxide were obtained from *Alfa Aesar* (96%). The telluric acid (98%) and the D_2_O (99%) used come from *Sigma-Aldrich* (98%). Phosphoric acid (purity of 85%) and sodium hydroxide (99%) from *Grüssinger* were used. Hydrochloric acid (37%) from *Carl-Roth* and sodium carbonate (99%), sodium acetate (99%), potassium perchlorate (98%), and ethanol (100%) were sourced from *Merck*.

### 2.2 POM Synthesis

The synthesis of the POMs used in this study is described in the corresponding section of the Supporting Information.^35–39^

### 2.3 POM Characterization

The stoichiometry of the synthesized POMs was verified by optical emission spectrometry with inductively coupled plasma (ICP-OES) as well as thermogravimetric analysis (TGA) to determine the hydration water content. The sodium content was measured via flame atomic absorption spectroscopy (F-AAS).

Samples of the solid POMs and liquid solutions were submitted to the Central Elemental Analysis Service at the University of Hamburg for ICP-OES analysis. The liquid samples of the feed solution were diluted with water by a factor of 10 beforehand. The instrument used for this analysis was an ARCOS from SPECTRO. For F-AAS, the THERMO SCIENTIFIC-SOLAR S Series instrument was used.

The integrity of the synthesized POMs, as well as that of the recovered POMs after membrane separation, was verified using Attenuated Total Reflection Fourier-Transform Infrared Spectroscopy (ATR-FT-IR). To analyze the vibrational properties of the four types of POMs, solid powder IR spectra were acquired in the ATR measurement mode by using a SHIMADZU QATRTMS single reflection ATR equipped with a diamond prism. The obtained raw data were baseline-corrected, and the peaks were manually identified.

For the validation of the corresponding POM structure in solution, Raman spectroscopy was used, and the results were compared with the Raman spectra of the recovered POMs after membrane separation.

The Raman spectra for the liquids were measured by using a SENTERRA Raman microscope from BRUKER OPTIK GmbH. The aperture was set to 50 × 1000 μm. A 20x objective was used on the microscope. The excitation laser had a wavelength of 785 nm, and the measurement range used was between 75 and 1525 cm^–1^. The integration time was 16 s; the number of scans was eight, and the Raman laser power was 10 mW.

Finally, for the UV–vis measurements carried out, an AGILENT TECHNOLOGIES Cary 60 UV–vis spectrometer was used together with a quartz glass cuvette of 10 mm path length.

### 2.4 Experimental Setup and Typical Working Procedure

All experiments were performed using a custom-designed experimental setup, as described in a previous study.^25^ A detailed description of the setup can be found in the membrane setup section of the Supporting Information (see Figures S1 and S2). The membranes with an active layer of 33 cm^2^ were pretreated in accordance with the supplier’s instructions. In the standard procedure, the POM solution (100 mL) was filled into the feed tank all at once. The pump was then switched on, and a flow rate of 15 mL·min^–1^ was set. Once the solution had reached the membrane cell, the integrated stirrer was set to its maximum speed, and the process was operated for 5 min without pressure. During this period, no samples were taken, and all flows were recycled back to the feed vessel. After 5 min, the pressure could be adjusted between 20 and 35 bar automatically by a relief valve. Once stable operation has been established (after 15 min), samples can be taken. For parameter variation, the membrane was conditioned with the POM solution using the corresponding parameters.

### 2.5 Membranes

All used membranes were commercially available polymer-based membranes with polypiperazine as the separation layer. The DK-series membrane was purchased from *Veolia*. The XN-45 and UA60 were bought from MANN + HUMMEL. Finally, the TS-40 and TS-80 were donated samples from MANN + HUMMEL. An overview is added to the Supporting Information (see Table S1), and a detailed description of the membranes can be found in manufacture data sheets.^40–44^ The pure water permeability was measured for all membranes from 12 to 14 bar (Figure S3).

The scanning electron microscopy (SEM) images of the membranes were taken by the Electron Microscopy Department of the Scientific Service of the University of Hamburg using a LEO Gemini 1550 from ZEISS. The acceleration voltages can be found in the information bar of the images. Energy-dispersive X-ray spectroscopy (EDX) for the detection of POM residues was carried out with the same device.

### 2.6 Formulas for Calculation

The rejection (*R*_*x*_) for component *x* was calculated using (eq 1) and the measured mass concentration of component *x* in the permeate (β_*x*_) and feed (β_*x*_)­

1 $$R_{x}=1-\frac{\beta_{x}\left(\mathrm{permeate}\right)}{\beta_{x}\left(\mathrm{feed}\right)}$$

The mass concentration was measured via ICP-OES as described for POM characterization. The permeate flow (*ṁ*) was measured over time (*t*) until a volume of approximately 10 mL of permeate (*m*(*p*ermeate)) was collected (eq 2). To meet the membrane field standard, the data was converted to liters per membrane area per hour (LMH). The active area of the membrane (*a*) is 33 cm^2^.

2 $$ṁ=\frac{m\left(\mathrm{permeate}\right)}{t\cdot a}$$

## 3 Results and Discussion

### 3.1 Overview of the Synthesized POMs Used for the Development of Their Recovery Process

In this work, a systematic study of the recovery of four different types of POMs from aqueous solution via nanofiltration was conducted (Figure 1). The different POM-types differ in size from 0.83 nm for the isopolyoxometalate of the Lindqvist-type up to 1.3 nm for the heteropolyoxometalate of the Wells–Dawson-type, as well as in geometry with a “cubic” shaped Keggin-type and a “planar” shaped Anderson-Evans-type.^45^ Not only are the shape and size of the POMs different but also the charge of the respective anions as well. A hydrated metal ion has the size of approximately 0.19 to 0.24 nm for transition metals in aqueous solution.^46^

**Figure 1:**
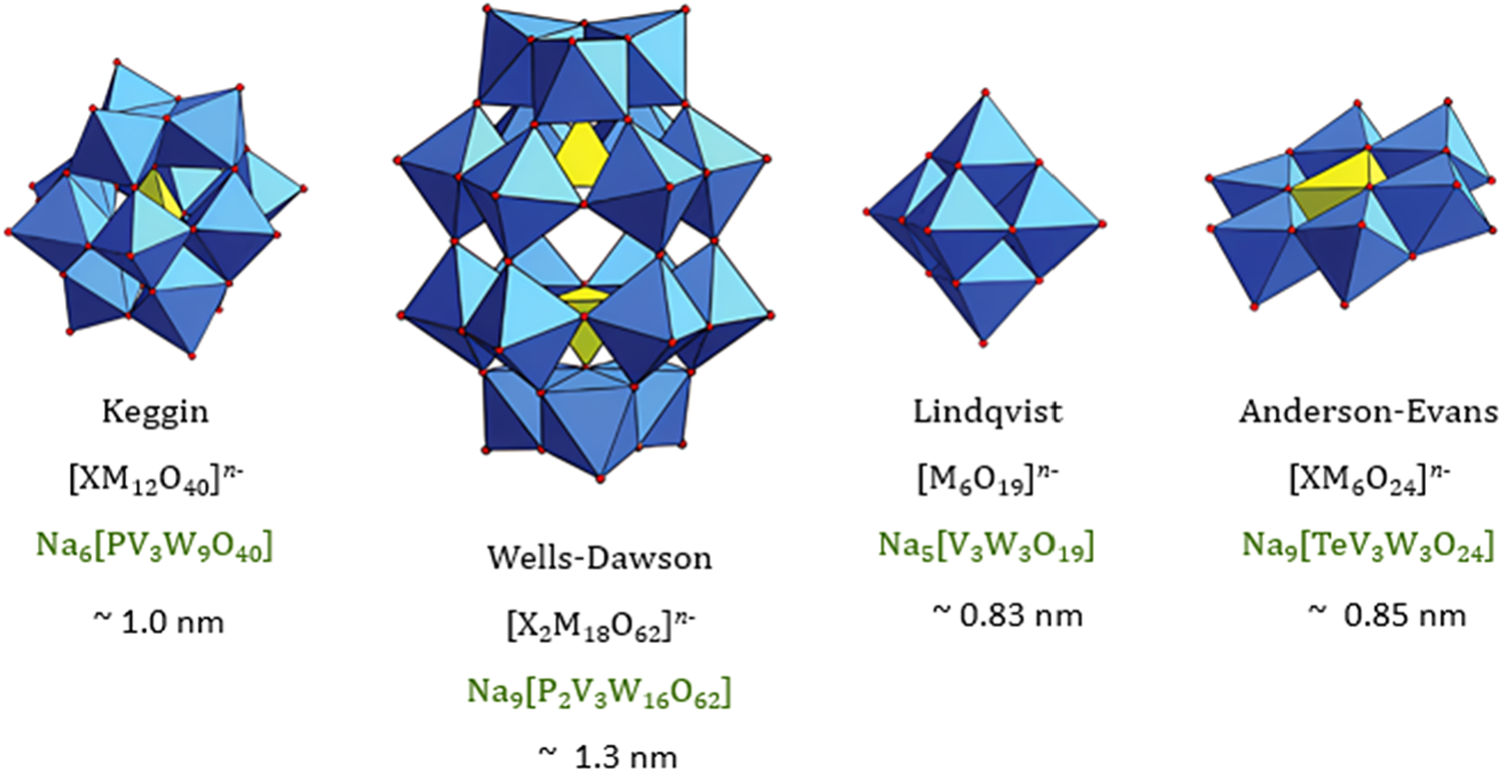
Schematic representation of the used polyoxometalates. Adapted with permission from Soria-Carrera, H. et.al. *Chemical Science* 2023, 14(1), 10–28. Copyright 2023 American Chemical Society.^47^

Since the Anderson-Evans-type cannot be synthesized with P as the center atom, tellurium (Te) was used instead.^33^ Crucial for the recovery in aqueous solutions of the different POMs is the maximum rejection of their components, such as P, Te, Tungsten (W), and V, as well as the stability of the POM structure during the downstream process. Therefore, these parameters are the most significant for evaluating the membrane process as a suitable purification method. The behavior of the membranes in terms of fouling and longevity was previously investigated in a separate study with Keggin-type POMs.^48^

### 3.2 Membrane Screening and Stability of the Different Polyoxometalates

#### 3.2.1 Lindqvist-Type POM

For the first evaluation, different commercial membranes were tested for the recovery of the various POM structures with a cut-off ranging from 150 to 1000 Da, starting with the Lindqvist-type POM in aqueous solution. The results are summarized in Table 1 and clearly show a high rejection for all four membranes with a cut-off until 500 Da with 99.2–99.6% for W and 97.1–99.2% for V. For the UA60 membrane, the V rejection decreased to 88% which indicates that the cut-off and therefore the pore size, here at 1000 Da, of the used membrane is important for an efficient rejection for the Lindqvist heteropolyanion. With 500 Da and lower, the pore size of the tested membranes is simply too small, and the Lindqvist POM is most likely rejected due to steric exclusion. The UA60 has an effective pore radius of 0.89 nm,^49^ which is slightly bigger compared to the size of the Lindqvist-type POM of 0.83 nm. The rejection of the catalyst is still relatively high, which indicates that other effects are also accountable for that, like the charge surface of the membrane. Indeed, the negatively charge membrane surface of the polypiperazine-amid separation layer at a pH value of 6 (feed),^50^ might play a role due to charge repulsion as POMs are multivalent anions.

**Table 1:** Rejection of the Metals Present in the Lindqvist Structure and Permeate Flow Rate for Different Membranes^a^

membrane	Cut-Off (Da)	tungsten(%)	vanadium (%)	permeate flow (L m^‑2^ h^‑1^)	effective pore radius of the membrane (nm)
TS-80	150–200	99	99	248	0.36–45 ^51,52^
DK-Series	150–300	99	99	206	0.46 ^53^
TS-40	200–300	99	99	223	0.51 ^54^
XN-45	300–500	99	97	260	0.53 ^53^
UA60	1000	97	88	303	0.89 ^51^

a *Experimental conditions*: prewetted membranes, ambient temperature, 5 mM Na_5_[V_3_W_3_O_19_], 100 mL of H_2_O, 15 mL·min^–1^ pump flow, *p* = 32 bar, 1100 rpm.

The permeate flow increases with the cut-off range from 248 L m^–2^ h^–1^ for the TS-80 to 303 L m^–2^ h^–1^ when UA60 is used.

Considering that the detection limit was reached for ICP-OES analysis, the UV/vis spectra of the permeate solution were additionally measured. In detail, the ligand-to-metal-charge-transfer bands (LMCT) of V^V^-substituted phosphotungstates could be observed, with the LMCT band at 198 nm corresponding to the LMCT from oxygen to W^VI^ (O → W^VI^) and the second band at 263 nm belonging to the LMCT of oxygen to V^V^ (O → V^V^).^55^ The lowest absorbance was measured for the permeate solution of the TS-80 membrane, where the highest absorbance was measured for the permeate solution of the XN-45 membrane (see Figure 2). The permeate solution of the UA60 membrane was not considered since the rejection was significantly lower. While the extinction depends on the concentration of the component, the latter is also correlated with the rejection of the POM.^56^ Again, the rejection of the Lindqvist-type POM increased with decreasing pore size of the membrane, driven by steric exclusion.

**Figure 2:**
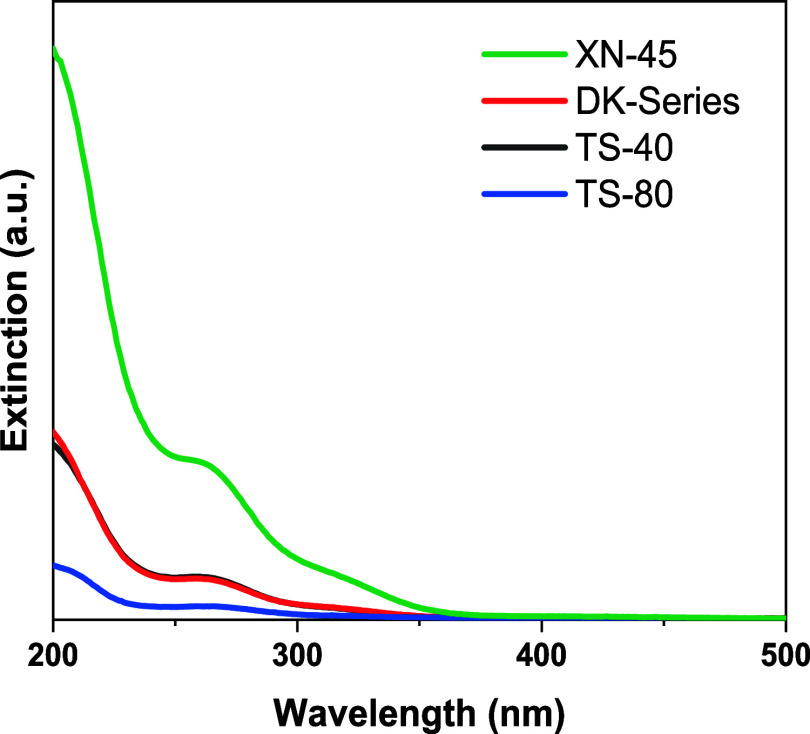
UV/vis spectra of undiluted permeate solution after the membrane separation of Na_5_[V_3_W_3_O_19_] for DK-Series (red), TS-40 (black), TS-80 (blue), and XN-45 (green).

It should be noted here that UV/Vis spectroscopy is ideal for the rapid analysis of membrane performance. With the appropriate extinction coefficients, even quantification is possible. This can be a significant advantage for operando analysis for future applications.

Even though the membrane separation is driven by physical processes, the catalyst might interact or even react with the membrane material.^57^ This could become apparent through a change in the catalyst. Consequently, to evaluate the integrity of the Lindqvist-type POM structure, another experiment with a higher concentration was performed using a TS-80 membrane. The rejection of the POM remained high with V and Te with 99%, and a decrease in permeate flow to 176 L m^–2^ h^–1^ was observed. The vibrational properties of the Lindqvist structure in solution were measured via Raman spectroscopy before and after membrane separation (see Figure 3). The signals at 998, 963, and 913 cm^–1^ could be assigned to the terminal metal–oxygen bonds (M=Ot) and the signals at 238 and 286 cm^–1^ to the edge-sharing metal–oxygen bonds (M=O_c_). The other signals in the Raman spectra belong to water. The solvent was evaporated and further analyzed by using IR spectroscopy and ICP-OES.

**Figure 3:**
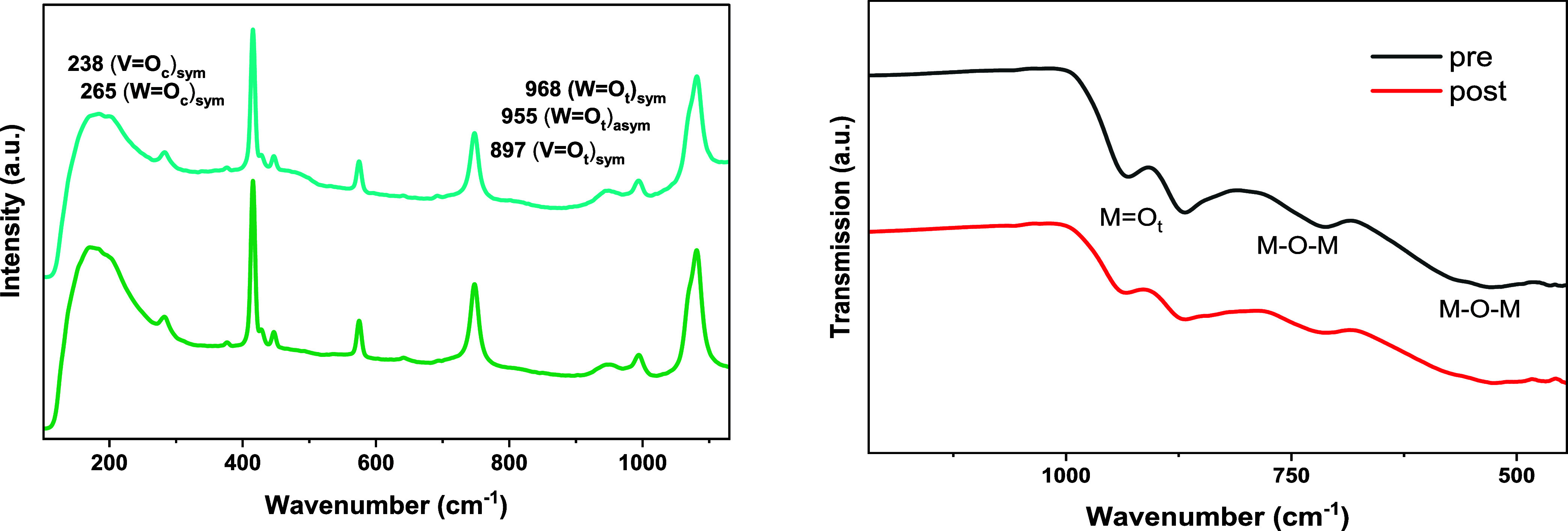
Left: Raman spectroscopic data of Na_5_[V_3_W_3_O_19_] in aqueous solution before (bottom) and after (top) membrane separation. Right: ATR-FT-IR spectra of solid Na_5_[V_3_W_3_O_19_] before (bottom) and after membrane separation (top). *Experimental conditions*: prewetted membranes, ambient temperature, 25 mM Na_5_[V_3_W_3_O_19_], 100 mL of H_2_O, 15 mL·min^–1^ pump flow, *p* = 32 bar, 1100 rpm.

Two bands at 934–935 cm^–1^ and 868 cm^–1^ could be assigned to the stretching modes of terminal oxygen atoms (M=O_t_). For the bridging oxygen atoms, the modes of M–O–M bonds appeared at 713 cm^–1^ and 526 cm^–1^.^37^ By comparing the IR and Raman data of the POMs before and after membrane separation, it could be revealed that all characteristic vibration bands for the Lindqvist-type structure were still present (see Figure S4). ICP-OES revealed a molecular composition for the synthesized compound with 2.9/3.0 V/W, which is comparable to 3.0/3.0 V/W after the membrane process. Additionally, no changes could be observed in the ^51^V spectra of the liquid solution before and after nanofiltration (see Figure S5). These results clearly demonstrate that the Lindqvist-type POM structure remained intact and that no leaching occurred. Therefore, it can be concluded that the Lindqvist-type POM does not undergo any structural changes during the membrane process and can be recovered with high efficiency. The limitation is implied by the cut-off range, where membranes with a cut-off of 500 Da or less are suitable for recovery, whereas larger pore sizes might fail to achieve full catalyst recovery under the tested conditions.

#### 3.2.3 Anderson-Evans-Type POM

To further investigate if the size or the charge of the POM has a dominant effect on POM recovery, the Anderson-Evans-type POM with a similar size and the same set of membranes was used. The main differences compared to the Lindqvist-type POM are the geometric shape and Te as a central heteroatom. As expected, the rejection rates for V and Te were high, with 99%. Again, the catalyst is too big to permeate through the membranes. For the UA60 membrane, the rejection for W and V decreased again for V to 95%. The Lindqvist structure has a size of 0.83 nm, whereas the Anderson-Evans structure has a size of 0.85 nm. The charge density per atom for [TeV_3_W_3_O_24_]^9–^ is 0.29 per atom, which is higher than for [V_3_W_3_O_19_]^5–^ with 0.20 per atom. The UA60 rejects less of the Lindqvist POM, which may be due to lower steric hindrance or a decrease in the charge density.

Surprisingly, the rejection of the heteroatom Te decreased with increasing cut-off from 98% to 19% (see Table 2). Analyzing the feed and permeate solutions with ICP-OES revealed that Te was lost during the membrane process. The feed solution had a molar ratio of 1/3/3 (Te/V/W), whereas the permeate solution from the XN-45 membrane had a ratio of 32/5/3, and the one from the TS-80 membrane had a ratio of 36/5/3. In the case of the UA60 membrane, the solution had a molecular ratio of 80/80/3. Clearly, Te passes through the membrane without being incorporated into the Anderson-Evans structure anymore with a size approximately between 0.19 and 0.24 nm.^46^ The feed solution had a pH value of 4.5, while the permeate solution had a value of 5.4 to 5.6 for all used membranes. The W-based Anders-Evans structure is known to be stable in a pH range of 4.0–7.5, and the feed solutions had a pH value of 5.^58^ Further, POMs are known for a complex dissociation behavior where various species exist in an equilibrium.^59^ This means that some of the Te may dissociate from the structure. During the recovery process, the metal ion is less rejected by the membrane, as it is most likely significantly smaller than the Anderson-Evans-type POM. For the TS-80, the rejection rate is relatively high at 98%, despite the significantly larger pore size. Therefore, charge effects may impact the exclusion of the Te metal ions. The dissociation behavior of V–W mixed Anderson-Evans-type POMs is generally not yet fully understood, which could be a focus for future research.

**Table 2:** Rejection of the Metals Present in the Anderson-Evans Structure and Permeate Flow Rate for Different Membranes^a^

membrane	cut-off (Da)	tellurium (%)	tungsten (%)	vanadium (%)	permeate flow (L m^‑2^ h^‑1^)	effective pore radius of the membrane (nm)
TS-80	150–200	98	99	99	227	0.36–45 ^53,54^
DK-Series	150–300	85	99	99	248	0.46 ^55^
TS-40	200–300	81	99	99	185	0.51 ^54^
XN-45	300–500	62	99	99	223	0.53 ^53^
UA60	1000	19	98	95	242	0.89 ^51^

a *Experimental conditions*: prewetted membranes, ambient temperature, 5 mM Na_9_[TeV_3_W_3_O_24_], 100 mL of H_2_O, 15 mL·min^–1^ pump flow, *p* = 32 bar, and 1100 rpm.

In contrast to the Lindqvist-type POM, the used membrane and pH value of the solution might be crucial for the recovery via nanofiltration. UV/vis spectra (see Figure S6) of the permeate solutions show the same trend as for the Lindqvist-type structure, where the XN-45 membrane leads to the lowest and the TS-80 membrane to the highest retention for V and W, respectively.

Since Te leaching has been observed, another experiment with a higher concentration was performed to investigate the integrity of the Anderson-type structure with the TS-80 membrane. Again, high rejection was obtained: 99% for V and W and 98% for Te, respectively. Raman and IR spectra were collected from the liquid solutions and solids after the solvent had been evaporated, and the spectra were compared with each other (see Figures 4 and S7). Surprisingly, the characteristic vibrational bands for the Anderson-Evans structure are still intact, and the molecular composition of the POM after the membrane process with 0.8/2.8/3 Te/V/W did not change as compared to before 0.8/2.8/3.0 Te/V/W. The ^51^V NMR spectra of the liquid solutions confirmed that the Anderson-Evans structure is still intact (see Figure S8). Nevertheless, Te leaching was observed even for the TS-80 membrane under the process conditions, which makes this recovery process unsuitable in terms of long process time.

**Figure 4:**
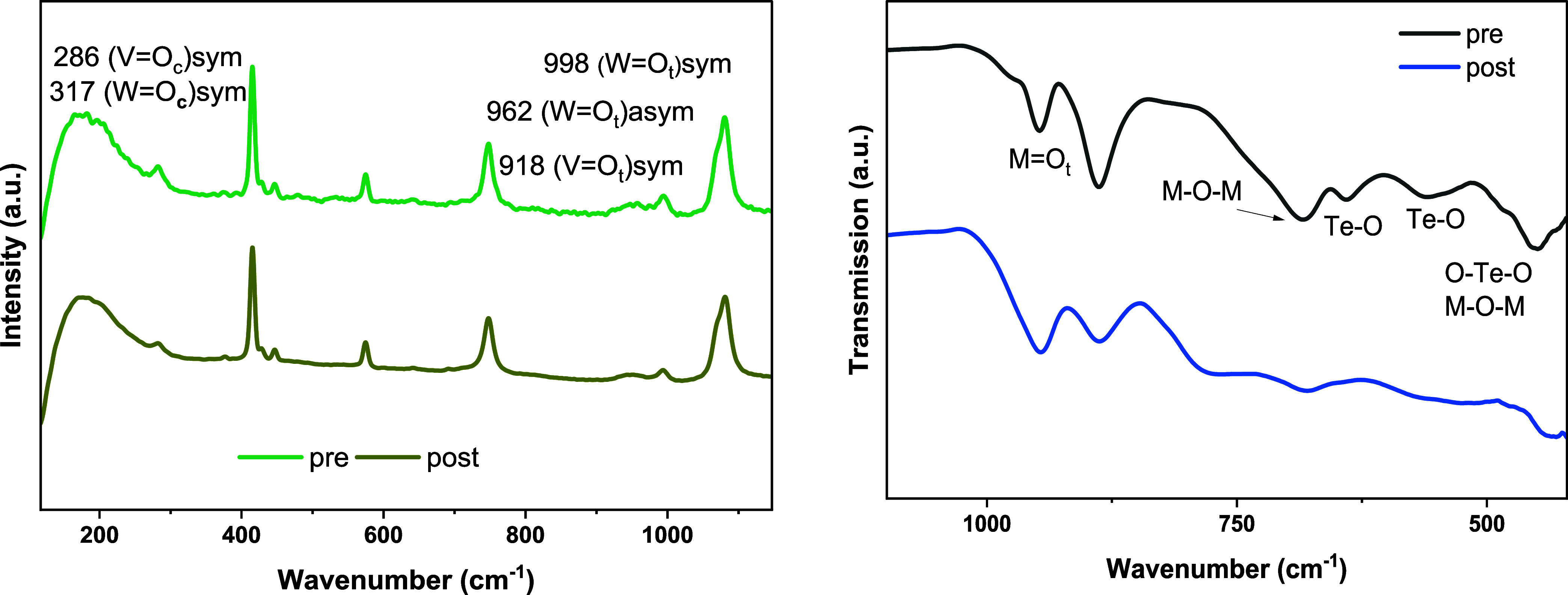
Left: Raman spectroscopic data of Na_9_[TeV_3_W_3_O_24_] in aqueous solution before (bottom) and after (top) membrane separation. Right: ATR-FT-IR spectra of solid Na_9_[TeV_3_W_3_O_24_] before (bottom) and after membrane separation (top). *Experimental conditions*: prewetted membranes, ambient temperature, 25 mM Na_9_[TeV_3_W_3_O_24_], 100 mL of H_2_O, 15 mL·min^–1^ pump flow, *p* = 32 bar, and 1100 rpm.

#### 3.2.4 Keggin and Wells–Dawson POM

To investigate the effect of an increased POM size on recovery, the same type of membranes with Keggin-type (1.0 nm) and Wells–Dawson-type (1.3 nm) POMs were used. The rejection for all elements was 99% for both POMs and all membranes except the UA60 membrane (see Tables S2 and S3). Probably the larger POMs are rejected via steric exclusion. In the UV/vis spectra (Figure 5), the TS-80 membrane had the highest retention for both of the structure types. This is in good agreement with previous studies, where a rejection of 99% was reached for V and Mo, as well as for the XN-45 membrane, or >99% for the DK-Series.^25^ The rejection of P was significantly lower in the former study, at >89% for the DK and XN-45 membrane. This deviation was probably caused by excessive dilution during sample preparation for ICP-OES.

**Figure 5:**
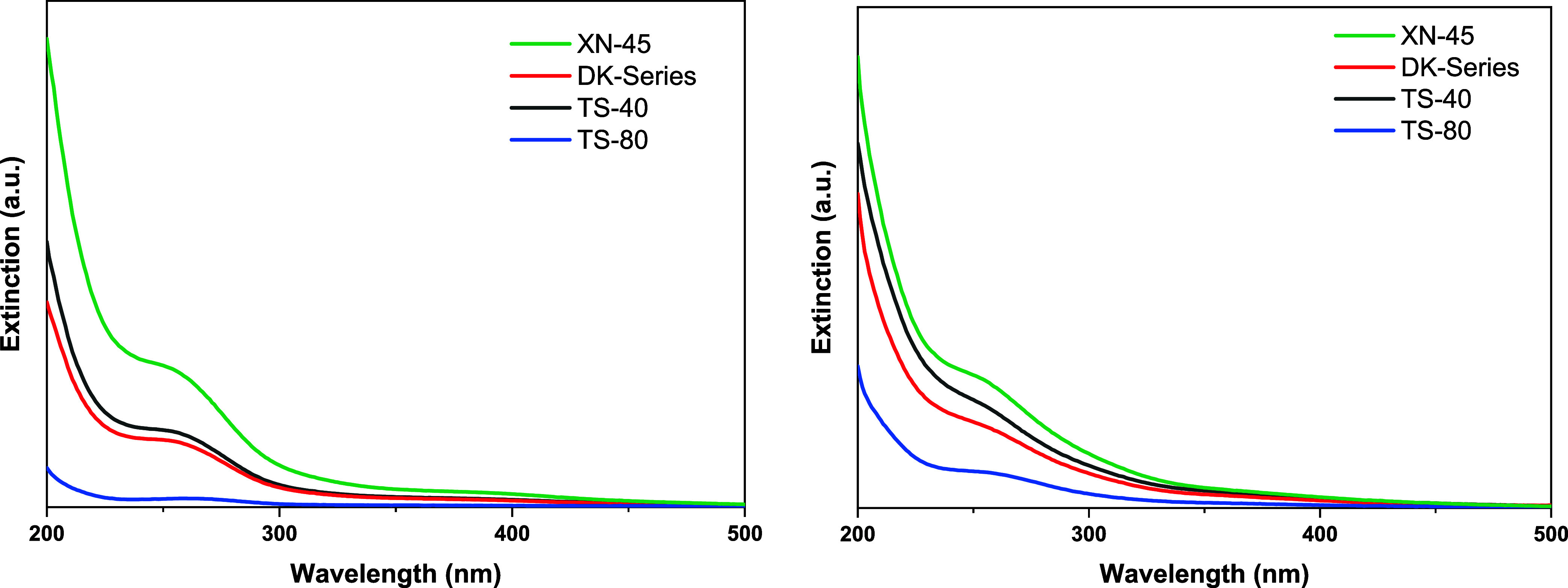
UV/vis spectra of undiluted permeate solutions after the membrane separation of Na_6_[PV_3_W_9_O_40_] (left) and Na_9_[P_2_V_3_W_15_O_62_] (right) for DK-Series (black), TS-40 (red), TS-80 (blue), and XN-45 (green).

In this study, the permeate solutions were not diluted further, and therefore, it was possible to increase the detection limit. For the UA60 membrane, the rejection of P for both POMs were 96% for V and 99% for W, respectively. Therefore, only a small fraction of the heteroatoms passed through the UA60 membrane, which may be due to the excess amount of P used during synthesis. Compared to the Lindqvist-type and Anders-Evans-type POMs, we observed an effect of the cut-off of the membrane on the rejection of the POM-type and, therefore, most likely of the size. However, it could be proven by Raman spectroscopy (Figure S9), FT-IR (Figure S10), as well as ^51^V and ^31^P NMR spectroscopy (Figures S11,S12) that the Keggin structure remained intact. For the Wells–Dawson-type POM, the experiment was repeated with a higher POM concentration, where the rejection of all elements remained at 99%. As can be seen in the Raman and FT-IR spectra, the structure of the Wells–Dawson-type POM remained intact (Figures 6 and S13).^60^ No changes were observed in the NMR spectra either (Figures S14,S15).

**Figure 6:**
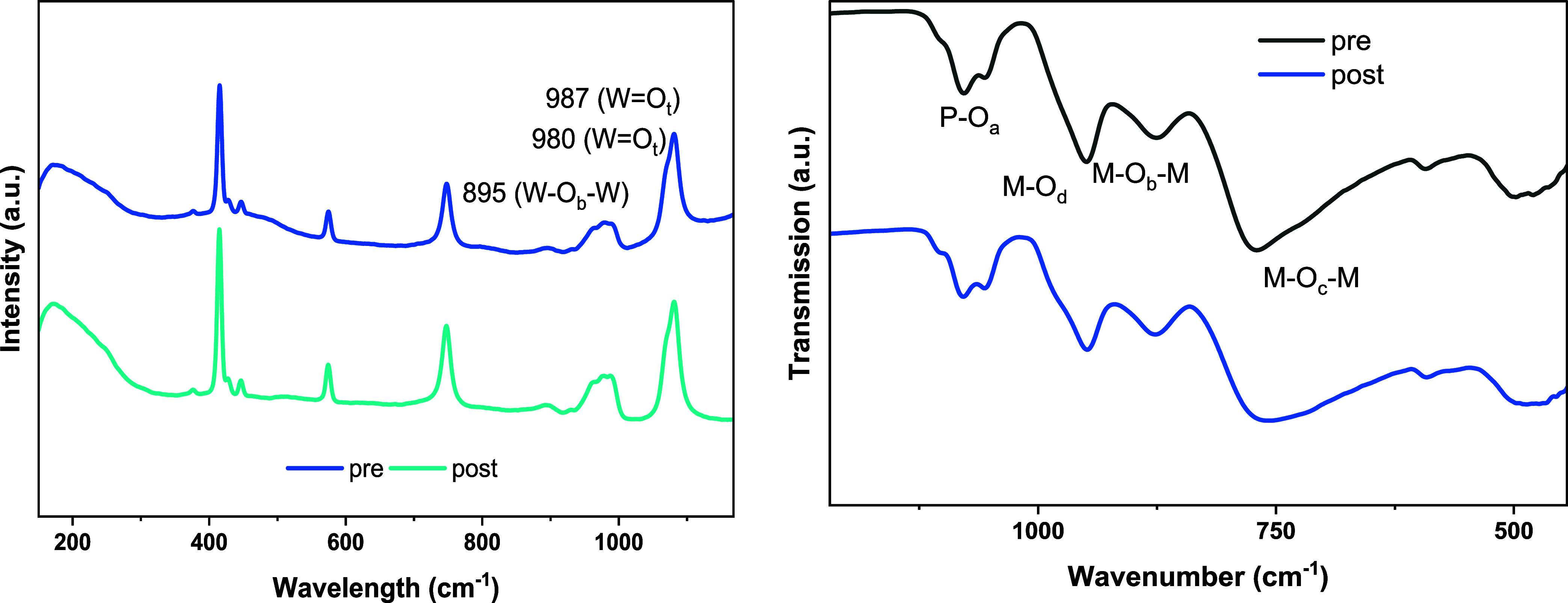
Left: Raman spectroscopic data of Na_9_[P_2_V_3_W_15_O_62_], in aqueous solution before (bottom) and after (top) membrane separation. Right: ATR-FT-IR spectra of solid Na_9_[P_2_V_3_W_16_O_62_] before (bottom) and after membrane separation (top). *Experimental conditions*: prewetted membranes, ambient temperature, 25 mM Na_9_[P_2_V_3_W_15_O_62_], 100 mL of H_2_O, 15 mL·min^–1^ pump flow, *p* = 32 bar, and 1100 rpm.

In contrast to the Anders-Evans-type, the heteroatom P did not leach through the membrane when the Wells–Dawson-type POM was used because it is incorporated into the structure and cannot dissociate without significantly altering the POM structure. The molecular composition measured via ICP-OES of the solids before 1.8/3/15 P/V/W and after 1.8/3/15 P/V/W confirmed the integrity of the Well-Dawson-type structure, indicating that the POM was completely recovered.

Taking the results of the investigated POMs into account, the main exclusion mechanism is steric exclusion, especially when membranes with small pore sizes are used. However, once the pore size matches the POM sizes, charge effects such as dielectric or Donnan exclusion may have a significant impact (Figure 7). The zeta potential of the membranes used ranges from −7 to −27 mV at a pH value of 7, and their isoelectric points range from 2.5 to 4.5 (Table S1). Depending on the structure type, the pH value of the feed solution ranges from 4.5 to 7.^61,62^ Under separation conditions, the surface of the membrane is always negatively charged and could reject the negatively charged POM by Donnan exclusion, where the positively charged counterions are attracted by the membrane surface. This effect is observable in the case of purification of the POMs during synthesis, where alkali salt separation was reached with high efficiency.^26^

**Figure 7:**
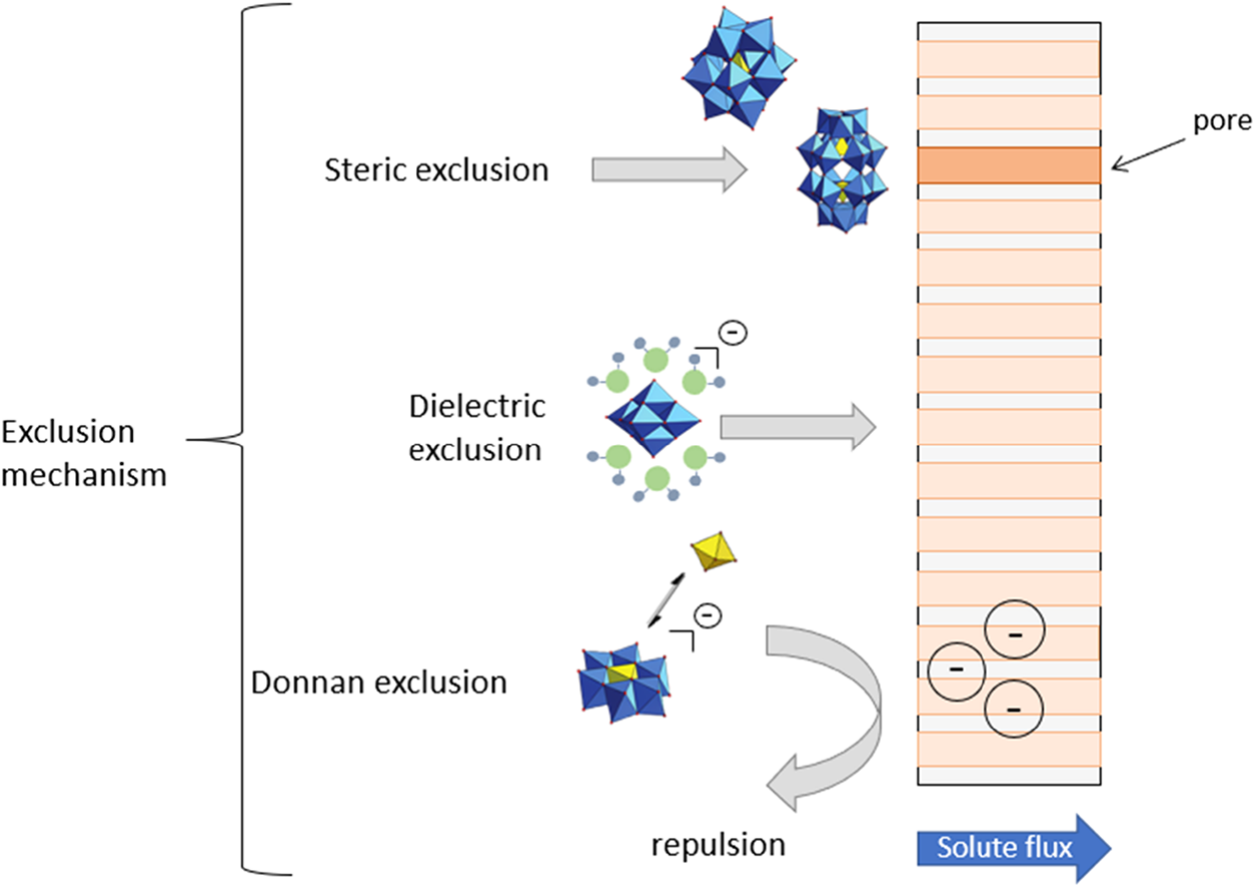
Schematic representation of the exclusion mechanism of POMs via nanofiltration.

Dielectric exclusion might play an important role, whereby the hydration shell of the POMs is considered. The reported sizes of different POM structures were taken from their respective crystal structures,^63^ unfortunately the hydrodynamic radii for the used POMs are not reported in the literature. Nevertheless, the charge density for the smaller POMs is relatively high compared to [PV_3_W_9_O_40_]^−6^ with 0.11 per atom and to [P_2_V_3_W_15_O_62_]^−9^ with 0.10 per atom, which could be another reason for the high rejection of the anions. Furthermore, for a complete description of the exclusion mechanisms, as in the case of the Anderson-Evans POM, the dissociation behavior of the POMs must be considered, especially since speciation is pH-dependent.

### 3.3 Impact of Separation Parameters in the Membrane Setup

#### 3.3.1 Impact of POM Concentration

To gain a better understanding of the parameters that influence the retention of the POMs, the concentration was varied between 1 and 50 mM of the stock solution for each POM-type. The TS-80 membrane had the highest rejection for all POM structures, and therefore, this membrane was used in the following section. As the concentration of the stock solution increased, a higher retention for Lindqvist- and Anderson-Evans-type POMs was observed at higher feed concentrations. A rejection of 99% both for V and W was achieved from a feed concentration of 5 mM Na_5_[V_3_W_3_O_19_] (Table S4). Very similar was the rejection of V and W for the Anderson-Evans-type POM (Table 3). The retention with a 1 mM feed solution of Na_9_[TeV_3_W_3_O_24_] was 97% for V and 98% for W, respectively. In contrast, a large dependence of the concentration on Te retention was observed. Starting with 90% at 1 mM and reaching its maximum with 98% at 50 mM of Na_9_[TeV_3_W_3_O_24_] in the solution. The Te leaching was relatively higher for lower concentrations than for higher concentrations. We suggest that with increasing feed concentrations, the size of the diffusing layer increases because of concentration-side polarization.^64,65^ Very often, this leads to a lower solute rejection rate, which is attributed to the attenuation of electrostatic exclusion and due to an increased osmotic pressure.^65^ On the other hand, the accumulation of the POM near the surface area might sterically or physically block the Te ions from passing through the membrane.^66^ This could result in a higher rejection rate but in a lower permeate flow as the net driving force for water transport declines.

**Table 3:** Rejection of the Metals Present in the Anderson-Evans Structure for Different Concentrations and the Measured Permeate Flow Rate^a^

feed concentration (mM)	tellurium(%)	tungsten (%)	vanadium (%)	permeate flow (L m^‑2^ h^‑1^)	permeate composition
1	90	98	96	232	38/5/3
5	96	99	99	210	11/3/3
10	97	99	99	198	7/3/3
25	96	99	99	173	4/3/3
50	98	99	99	147	3/3/3

a *Experimental conditions*: prewetted TS-80 membrane, ambient temperature, Na_9_[TeV_3_W_3_O_24_], 100 mL of H_2_O, 15 mL·min^–1^ pump flow, *p* = 32 bar, and 1100 rpm.

The same effect could also be assumed for the Lindqvist-type POM, because complete retention only occurred from a feed concentration of 5 mM, and the permeate flow also decreased significantly from 232 L m^–2^ h^–1^ for 1 mM to 147 L m^–2^ h^–1^ for 50 mM Na_5_[V_3_W_3_O_19_]. In contrast, the Keggin-type and Wells–Dawson-type POMs were rejected almost completely at low feed concentration, with 99% rejection for all elements (Tables S5 and S6). Again, assuming the rejection of the different POMs depends mainly on the size of the corresponding structure type, the Keggin and Well-Dawson POMs were rejected because of their size, whereas the smaller types were rejected additionally due to charge effects accompanied by concentration-dependent polarization effects at feed concentrations >5 mM. A rejection of 99% for all elements could not be reached at concentrations below 5 mM. The color intensity of the permeate solutions increased with increasing feed concentration for all structure types. Even if 99% of the POM was rejected, the absolute concentration of the permeating POM increased as more ions might permeate the membrane due to an increase in osmotic pressure, leading to a color intensification (Figures S16–S19). This effect was observed for all structure types, independent of size or charge. Future processes should take this effect into account to avoid multiple separations or exceeding the desired amount of impurity.

Interestingly, the permeate flow rate varied differently depending on the structure type (Figure 8). At a feed concentration of 50 mM, the permeate flow was 120 L m^–2^ h^–1^ for Na_6_[PV_3_W_9_O_40_] and 91 L m^–2^ h^–1^ for Na_9_[P_2_V_3_W_15_O_62_]. The size of the diffusion layer not only depends on the concentration of the rejected component but also on its hydraulic diameter and, consequently, its size and shape.^63,66^ Therefore, we assume that the permeate flow decreases as the POM size increases. The charge density of the POM does not correlate with the flux decline, so it is less likely to have an influence. For feed concentrations of 1–5 mM, there was no clear observable effect.

**Figure 8:**
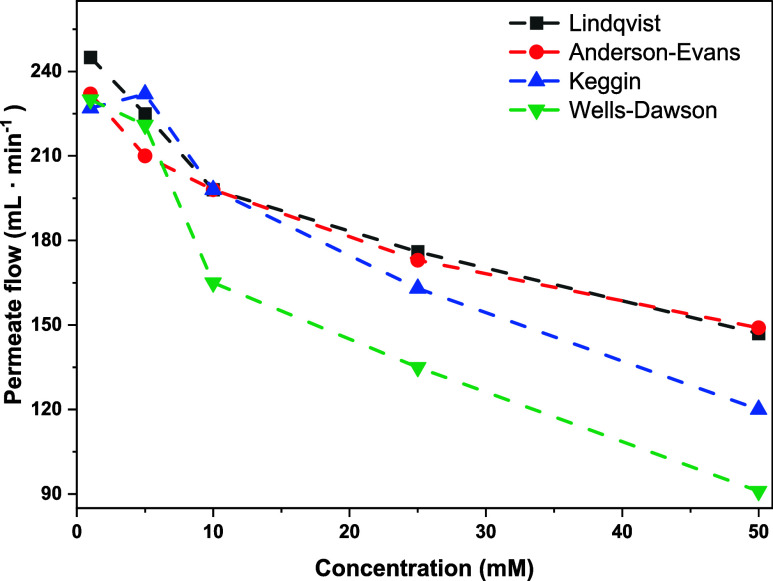
Permeate flow as a function of concentration and POM structure. For Lindqvist-type (black), Anderson-Evan-type (red), Keggin-type (blue), and Wells–Dawson-type (green). *Experimental conditions*: prewetted TS-80 membrane, ambient temperature, 100 mL of H_2_O, 15 mL·min^–1^ pump flow, *p* = 32 bar, and 1100 rpm.

In contrast to what has been described previously at this concentration, the diffusion layer may not impact the permeate flow or rejection, at least for smaller POMs such as the Lindqvist and Anderson-Evans structures. In addition, the permeate flow for low concentrations was quite fast, where inaccurate time measurements could cause fluctuations. In summary, the increased concentration leads in this case to an increase in solute rejection but to a decline in flux, which affects the efficiency.

Finally, the membranes used for each POM were analyzed after the concentration variation with SEM in order to ascertain whether any alterations to the membranes could be detected. Prior to measurement, all membranes were meticulously cleaned with water and subsequently dried. The membrane surface structure remained constant during the membrane separation process (Figures S20–S24). Occasional residues could be recognized, which could not be further determined by EDX. The membranes were therefore studied again using ICP-OES. The membranes were washed overnight with water on a shaking plate, and the washed residue was analyzed with ICP-OES. In the case of the Anderson-Evans-type POM, the measured metal concentration was below the quantification limit of 0.26 mg·L^–1^. For the Lindqvist-type, Keggin-type, and Wells–Dawson-type POMs, the concentration for W was <0.01% compared to the feed concentration used and for *P* < 0.02%, respectively. The residues observed on all membranes were found to be at a very low level, thus indicating the membrane’s potential for effective recovery of the POM. The cleaning of the membranes can easily be done with an aqueous solution. However, the investigation could also extend to the question of long-term stability.

#### 3.3.2 Impact of Flow and Pressure

For the Anderson-Evans-type POM, the pressure and flow rate were varied using the TS-80 membrane (Table S6 and Figure 9). In the case of the pressure enhancement, the permeate flow increased, as well from 103 to 182 L m^–2^ h^–1^, where the rejection of the POM was not affected significantly. In contrast, with a higher flow rate, the permeate flow was not affected as much with an increase of 182 to 191 L m^–2^ h^–1^. This clearly shows that pressure is the dominating effect here. A minor decrease in Te rejection was observed from 99.1 to 98.6% with increasing pressure, meaning increasing driving force.

**Figure 9:**
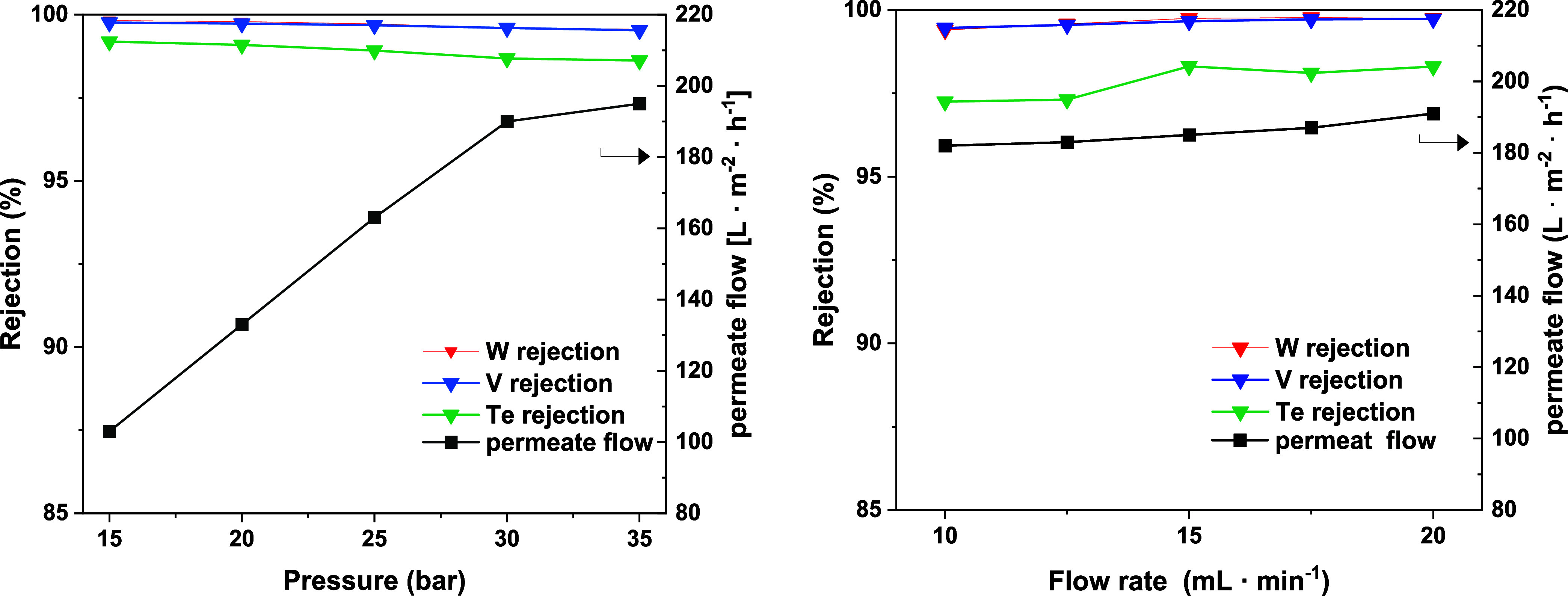
Rejection and permeate flow rate of the Anderson-Evans-type (black) metals as a function of pressure (left) and flow rate (right). Rejection of Te (green), W (red), and V (blue). *Experimental conditions*: prewetted TS-80 membrane, ambient temperature, 100 mL of H_2_O, 5 mM Na_9_[TeV_3_W_3_O_24_], 10–20 mL·min^–1^ pump flow, *p* = 15–35 bar, and 1100 rpm.

The rejection of V and W consistently remained at a high rate of 99%, while for Te, a higher rejection at 15 mL·min^–1^ with 98.2% compared to 97.3% at 10 mL·min^–1^ was observed. This could be due to a decrease in feed-side polarization, which reduces the osmotic pressure and increases the permeate flow rate. By maximizing pressure and flow rate, an average rejection for Te of 98.9% with a standard deviation of 0.2% was reached as determined by three experiments and an average permeate flow rate of 266 L m^–2^ h^–1^ with a deviation of 4 L m^–2^ h^–1^ (Table S7).

In summary, the TS-80 membrane, in conjunction with other membranes, has been demonstrated to be a viable option for the recovery of POMs in aqueous solutions. This efficacy was maintained under a range of process conditions and across a spectrum of concentrations with only a marginal decline in performance and stability. It is only in the case of the Anderson-Evans-type structure that further investigations might be required in order to avoid leaching of Te within the process. Modeling and further mechanistic investigation are conceivable in order to understand the behavior of POMs in the course of nanofiltration. Long-term studies and real-process solutions from catalytic applications are particularly relevant to future applications.

## 4 Conclusion

Nanofiltration represents a further development in the recovery of POMs from aqueous solutions. Several commercial polymer membranes, such as XN-45, DK-series, TS-40, TS-80, and UA60, could be used to recover Lindqvist, Anderson-Evans, Keggin, and Wells–Dawson-type POMs with rejections of almost 99%. However, with the UA60 membrane, only the large-sized Keggin- and Wells–Dawson-types could be retained almost completely. Steric exclusion appears to be the most important retention mechanism, although charge effects cannot be ruled out either. Raman spectroscopy, FT-IR, NMR, and ICP-OES analysis clearly demonstrate that the integrity of the POMs was maintained before and after the membrane separation process. The Anderson-Evans POM is an exception: ICP-OES measurements revealed slight Te leaching. Only a slight P leaching was detected for the UA60 membrane for Keggin- and Wells–Dawson-type POMs. Permeate flow was measured at varying feed solution concentrations; the different properties can lead to different permeate flows and, in some cases, different component retention. In the case of the Anderson-Evans-type POM, the variation in the flow rate and pressure had only a minor influence, whereby the size exclusion of the membrane itself had the greatest influence on the separation performance. Maximizing the flow rate and pressure resulted in a Te retention of almost 98.9%. For all other POM structures, the recovery was exceptionally high (>99%) while maintaining structural integrity. Consequently, the use of polymer membranes with different size exclusion properties offers a promising approach for downstream biomass-derived process solutions, and further research focusing on product separation is in progress.

## Supplementary Material



## Data Availability

Data will be made available on Zenodo after publication via http://dx.doi.org/10.5281/zenodo.17961026.

## Notes

Materials and methods; experimental setup and procedure; ^1^H, ^51^V, ^31^P NMR, and Raman spectra, SEM images; and tables with experimental results (PDF)

## References

[1] Moghadasi M., Abbasi M., Mousavi M., Mirzaei M. (2025). Polyoxometalate-Based
Materials in Therapeutic and Biomedical Applications: Current Status
and Perspective. Dalton Trans..

[2] Lin S., Li J., Zhou F., Tan B. K., Zheng B., Hu J. (2023). K6 [P2Mo18O62]
as DNase-Mimetic Artificial Nucleases to Promote Extracellular Deoxyribonucleic
Acid Degradation in Bacterial Biofilms. ACS
Omega.

[3] Zheng M., Ding Y., Cao X., Tian T., Lin J. (2018). Homogeneous
and Heterogeneous Photocatalytic Water Oxidation by Polyoxometalates
Containing the Most Earth-Abundant Transition Metal, Iron. Appl. Catal., B.

[4] Kikkawa S., Fujiki Y., Chudatemiya V., Nagakari H., Shibusawa K., Hirayama J., Nakatani N., Yamazoe S. (2024). Water-Tolerant Superbase
Polyoxometalate [H _2_ (Nb _6_ O _19_)] ^6–^ for Homogeneous Catalysis. Angew. Chem., Int. Ed..

[5] Miras H. N., Yan J., Long D.-L., Cronin L. (2012). Engineering Polyoxometalates with
Emergent Properties. Chem. Soc. Rev..

[6] López X., Carbó J. J., Bo C., Poblet J. M. (2012). Structure, Properties
and Reactivity of Polyoxometalates: A Theoretical Perspective. Chem. Soc. Rev..

[7] Guo H., Yin G. (2011). Catalytic Aerobic Oxidation
of Renewable Furfural with Phosphomolybdic
Acid Catalyst: An Alternative Route to Maleic Acid. J. Phys. Chem. C.

[8] Voitl T., von Rohr R. (2008). Oxidation of Lignin
Using Aqueous Polyoxometalates
in the Presence of Alcohols. ChemSusChem.

[9] Albert J., Wölfel R., Bösmann A., Wasserscheid P. (2012). Selective
oxidation of complex, water-insoluble biomass to formic acid using
additives as reaction accelerators. Energy Environ.
Sci..

[10] Reichert J., Brunner B., Jess A., Wasserscheid P., Albert J. (2015). Biomass Oxidation to Formic Acid in Aqueous Media Using
Polyoxometalate Catalysts - Boosting FA Selectivity by in-Situ Extraction. Energy Environ. Sci..

[11] Maerten S., Kumpidet C., Voß D., Bukowski A., Wasserscheid P., Albert J. (2020). Glucose Oxidation to
Formic Acid and Methyl Formate
in Perfect Selectivity. Green Chem..

[12] Voß D., Kahl M., Albert J. (2020). Continuous
Production of Formic Acid
from Biomass in a Three-Phase Liquid-Liquid-Gas Reaction Process. ACS Sustainable Chem. Eng..

[13] Okada T., Miyamoto K., Sakai T., Mishima S. (2014). Encapsulation of a
Polyoxometalate into an Organosilica Microcapsule for Highly Active
Solid Acid Catalysis. ACS Catal..

[14] Evtushok V. Yu., Lopatkin V. A., Podyacheva O. Yu., Kholdeeva O. A. (2022). Immobilization
of Polyoxometalates on Carbon Nanotubes: Tuning Catalyst Activity,
Selectivity and Stability in H2O2-Based Oxidations. Catalysts.

[15] Cherevan A. S., Nandan S. P., Roger I., Liu R., Streb C., Eder D. (2020). Polyoxometalates on Functional Substrates: Concepts, Synergies, and
Future Perspectives. Adv. Sci..

[16] Utievskyi Y., Neumann C., Sindlinger J., Schutjajew K., Oschatz M., Turchanin A., Ueberschaar N., Schacher F. H. (2023). Polyoxometalate-Modified Amphiphilic
Polystyrene-Block-Poly­(2-(Dimethylamino)­Ethyl
Methacrylate) Membranes for Heterogeneous Glucose to Formic Acid Methyl
Ester Oxidation. Nanomaterials.

[17] Wesner A., Papajewski M. P., Schidowski L., Ruhmlieb C., Poller M. J., Albert J. (2024). Supported
H 8 PV 5 Mo 7 O 40 on activated carbon: Synthesis
and Investigation of influencing factors for catalytic performance. Dalton Trans..

[18] Yan K., Yu Z., Wang Y., Guo M., Xiong J., Zhang R., Li X., Lu X. (2025). Polyoxometalates@
Metal–Organic Frameworks:
Synthesis Strategies, Nanostructure Modulation and Application in
Biomass Conversion. ChemSusChem.

[19] Veith H., Voges M., Held C., Albert J. (2017). Measuring and predicting
the extraction behavior of biogenic formic acid in biphasic aqueous/organic
reaction mixtures. ACS Omega.

[20] Jessop P. G. (2011). Searching
for Green Solvents. Green Chem..

[21] Osman A. I., Chen Z., Elgarahy A. M., Farghali M., Mohamed I. M. A., Priya A. K., Hawash H. B., Yap P. (2024). Membrane Technology
for Energy Saving: Principles, Techniques, Applications, Challenges,
and Prospects. Adv. Energy Sustainability Res..

[22] Abed K. M., Hayyan A., Hizaddin H. F., Hashim M. A., Basirun W. J., Saleh J., Hashim N. A. (2025). Superiority
of Liquid Membrane-Based
Purification Techniques in Biodiesel Downstream Processing. Renewable Sustainable Energy Rev..

[23] Kahloul M., Chekir J., Hafiane A. (2019). Dye Removal
Using Keggin Polyoxometalates
Assisted Ultrafi Ltration: Characterization and UV Visible Study. Arch. Environ. Prot..

[24] Bertleff B., Goebel R., Claußnitzer J., Korth W., Skiborowski M., Wasserscheid P., Jess A., Albert J. (2018). Investigations on catalyst
stability and product isolation in the extractive oxidative desulfurization
of fuels using polyoxometalates and molecular oxygen. ChemCatChem.

[25] Esser T., Huber M., Voß D., Albert J. (2022). Development of an efficient
downstream process for product separation and catalyst recycling of
a homogeneous polyoxometalate catalyst by means of nanofiltration
membranes and design of experiments. Chem. Eng.
Res. Des..

[26] Raabe J. C., Esser T., Jameel F., Stein M., Albert J., Poller M. J. (2023). Study on the Incorporation of Various Elements into
the Keggin Lacunary-Type Phosphomolybdate [PMo9O34]­9– and Subsequent
Purification of the Polyoxometalates by Nanofiltration. Inorg. Chem. Front..

[27] Esser T., Huber M., Voß D., Albert J. (2022). Development of an Efficient
Downstream Process for Product Separation and Catalyst Recycling of
a Homogeneous Polyoxometalate Catalyst by Means of Nanofiltration
Membranes and Design of Experiments. Chem. Eng.
Res. Des..

[28] Voß D., Pickel H., Albert J. (2019). Improving the fractionated catalytic
oxidation of lignocellulosic biomass to formic acid and cellulose
by using design of experiments. ACS Sustainable
Chem. Eng..

[29] Albert J. (2017). Selective
oxidation of lignocellulosic biomass to formic acid and high-grade
cellulose using tailor-made polyoxometalate catalysts. Faraday Discuss..

[30] Ishikawa E., Kihara D., Togawa Y., Ookawa C. (2019). Cyclooctene Epoxidation
by Hydrogen Peroxide in the Presence of Vanadium-Substituted Lindqvist-Type
Polyoxotungstate [VW5O19]­3. Eur. J. Inorg. Chem..

[31] Pawlig A. H., You W., Poller M. J., Albert J. (2025). Systematic Determination of Structure-Activity-Selectivity
Correlations for the Catalytic Oxidation of Glucose to Acetic Acid
Depending on the Structure Type of the Used Polyoxometalate Catalyst. Chem. - Eur. J..

[32] Jiang S., Chu Q., Gao J., Shi J. (2025). Study on the performance optimization
and mechanism of Dawson-type PWV polyoxometalate catalyzed oxidative
depolymerization of lignin to aromatic monomers. Biomass Bioenergy.

[33] Raabe J. C., Esser T., Poller M. J., Albert J. (2024). Synthesis and Characterization
of V Substituted Anderson-Type Telluro-Molybdates and Tungstates for
Catalytic Oxidation of Furan Derivatives to Formic and Maleic Acid. Catal. Today.

[34] Yang D., Xie S., Wu D., Ding J., Shi E., An P., Dai S., Guo L., Hou Z. (2025). Efficient
Oxidative Cleavage of β-O-4
Linkage in Lignin Model Compounds Enabled by a Simple Anderson-Type
Polyoxometalate. Green Chem..

[35] Raabe J. C., Aceituno Cruz J., Albert J., Poller M. J. (2023). Comparative Spectroscopic
and Electrochemical Study of V­(V)-Substituted Keggin-Type Phosphomolybdates
and -Tungstates. Inorganics.

[36] Albert J., Mehler J., Tucher J., Kastner K., Streb C. (2016). One–step
synthesizable lindqvist– isopolyoxometalates as promising new
catalysts for selective conversion of glucose as a model substrate
for lignocellulosic biomass to formic acid. ChemistrySelect.

[37] Yerra S., Amanchi S. R., Das S. K. (2014). Synthesis
and Structural Characterization
of Lindqvist Type Mixed-Metal Cluster Anion [V2W4O19] 4– in
Discrete and Coordination Polymer Compounds. J. Mol. Struct..

[38] Mbomekallé I., Lu Y.-W., Keita B., Nadjo L. (2004). Simple, High Yield
and Reagent-Saving Synthesis of Pure α-K6P2W18O62 · 14H2O. Inorg. Chem. Commun..

[39] Domaille P. J., Watunya G. (1986). Synthesis and Tungsten-183 NMR Characterization
of
Vanadium-Substituted Polyoxometalates Based on B-Type Tungstophosphate
PW9O349-Precursors. Inorg. Chem..

[40] MANN+HUMMEL Water & Fluid Solutions GmbH . PRODUCT SPECIFICATION-TRISEP UA60 - Manufacturer Data Sheet 2021 https://water-membrane-solutions.mann-hummel.com/content/dam/lse-wfs/product-related-assets/data-sheets/UA60.pdf. (accessed July 29, 2025).

[41] MANN+HUMMEL Water & Fluid Solutions GmbH . PRODUCT SPECIFICATION-TRISEP UA60 - Manufacturer Data Sheet 2021 https://water-membrane-solutions.mannhummel.com/content/dam/lse-wfs/product-related-assets/data-sheets/UA60.pdf. (accessed July 29, 2025).

[42] MANN+HUMMEL Water & Fluid Solutions GmbH . PRODUCT SPECIFICATIONTRISEP TS-40 - Manufacturer Data Sheet 2021 https://Water-membrane-Solutions.MANN-HUMMEL.com/content/dam/lse-wfs/product-related-assets/data-sheets/TS40.pdf. (accessed July 29, 2025).

[43] SUEZ SA . PRODUCT SPECIFICATION - DK Series Manufacturer Data Sheet.. www.lenntech.comFax.

[44] MANN+HUMMEL Water & Fluid Solutions GmbH . PRODUCT SPECIFICATION-TRISEP XN-45 - Manufacturer Data Sheet. 2021.

[45] Mallick L., Chakraborty B. (2022). Ionic Γ-FeO­(OH)
Nanocrystal Stabilized by Small
Isopolymolybdate Clusters as Reactive Core for Water Oxidation. Chem. - Eur. J..

[46] Persson I. (2024). Structure
and Size of Complete Hydration Shells of Metal Ions and Inorganic
Anions in Aqueous Solution. Dalton Trans..

[47] Soria-Carrera H., Atrián-Blasco E., Martín-Rapún R., Mitchell S. G. (2022). Polyoxometalate–peptide
hybrid materials: from structure–property relationships to
applications. Chem. Sci..

[48] Esser T., Wassenberg A., Voß D., Albert J. (2024). Novel Insights into
the Recovery and Recyclability of Homogeneous Polyoxometalate Catalysts
Applying an Efficient Nanofiltration Process for the Selective Catalytic
Oxidation of Humins. Chem. Eng. Res. Des..

[49] Guo H., Wang X., Wang K., Liu S. (2025). Adsorption of Natural
Organic Matter and Divalent Cations onto/inside Loose Nanofiltration
Membranes: Implications for Drinking Water Treatment from Rejection
Selectivity Perspective. Water Res..

[50] Wang J., Zhao C., Wang T., Wu Z., Li X., Li J. (2016). Graphene Oxide Polypiperazine-Amide
Nanofiltration Membrane for Improving
Flux and Anti-Fouling in Water Purification. RSC Adv..

[51] Bargeman G., Westerink J. B., Manuhutu C. F. H., Kate A. (2015). ten. The Effect of
Membrane Characteristics on Nanofiltration Membrane Performance during
Processing of Practically Saturated Salt Solutions. J. Membr. Sci..

[52] Gautam A., Menkhaus T. J. (2014). Performance Evaluation and Fouling
Analysis for Reverse
Osmosis and Nanofiltration Membranes during Processing of Lignocellulosic
Biomass Hydrolysate. J. Membr. Sci..

[53] Straatsma J., Bargeman G., van der
Horst H. C., Wesselingh J. A. (2002). Can Nanofiltration
Be Fully Predicted by a Model?. J. Membr. Sci..

[54] Yamase T. (1998). Photo-and
Electrochromism of Polyoxometalates and Related Materials. Chem. Rev..

[55] Song I. K., Kim H. S., Chun M.-S. (2003). On the Reduction Potential of Cation-Exchanged
Heteropolyacids (HPAs). Korean J. Chem. Eng..

[56] Rodger, A. Concentration Determination Using Beer-Lambert Law. In Encyclopedia of Biophysics; Springer, 2013; pp 360–361.

[57] Suhalim N. S., Kasim N., Mahmoudi E., Shamsudin I. J., Mohammad A. W., Mohamed Zuki F., Jamari N. L.-A. (2022). Rejection Mechanism
of Ionic Solute Removal by Nanofiltration Membranes: An Overview. Nanomaterials.

[58] Gumerova N. I., Rompel A. (2023). Speciation Atlas of
Polyoxometalates in Aqueous Solutions. Sci.
Adv..

[59] Gumerova N. I., Rompel A. (2020). Polyoxometalates in Solution: Speciation under Spotlight. Chem. Soc. Rev..

[60] Ueda T., Nishimoto Y., Saito R., Ohnishi M., Nambu J. (2015). Vanadium (V)-Substitution
Reactions of Wells–Dawson-Type Polyoxometalates: From [X2M18O62]
6–(X= P, As; M= Mo, W) to [X2VM17O62] 7. Inorganics.

[61] Mandale S., Jones M. (2008). Interaction of electrolytes and non-electrolytes
in nanofiltration. Desalination.

[62] żyłła R., Foszpańczyk M., Kamińska I., Kudzin M., Balcerzak J., Ledakowicz S. (2022). Impact of Polymer Membrane Properties on the Removal
of Pharmaceuticals. Membranes.

[63] Song Y., Qin W., Li T., Hu Q., Gao C. (2018). The Role of Nanofiltration
Membrane Surface Charge on the Scale-Prone Ions Concentration Polarization
for Low or Medium Saline Water Softening. Desalination.

[64] Shang C., Xia J., Sun L., Lipscomb G. G., Zhang S. (2022). Concentration Polarization
on Surface Patterned Membranes. AIChE J..

[65] Mahlangu O. T., Mamba B. B. (2025). Influence of Membrane
Salt Rejection Properties on
Cake-Enhanced Concentration Polarization Effects During Colloidal
Fouling of Nanofiltration Membranes. Membranes.

[66] Liu S., Foo Z., Lienhard J. H., Keten S., Lueptow R. M. (2025). Membrane Charge
Effects on Solute Transport in Nanofiltration: Experiments and Molecular
Dynamics Simulations. Membranes.

