# Supplementary material for: Investigations on the Recovery of Different Structure Types of Polyoxometalates from Aqueous Solution Using Nanofiltration Membranes
Source: ACS Omega. 2026 Apr 4;11(15):23290–301. doi: 10.1021/acsomega.6c00086 (PMC13103803; doi:10.1021/acsomega.6c00086)
Supplement: Supplementary file 1 [file ao6c00086_si_001.pdf]

Supporting information for

Investigations on the recovery of different structure types of polyoxometalates from aqueous solution using nanofiltration membranes

Leon Schidowski, Luca F. Friedrich, Andreas H. Pawlig, Dorothea Voß, Jakob Albert[*]

Institute for Technical and Macromolecular Chemistry, University of Hamburg, 20146 Hamburg, Germany

Content

1. Material and Methods 2
   1.1 Catalyst characterization methods 2
   1.2 Synthesis of catalysts 2
      1.2.1 Synthesis of Lindqvist-Type $Na_5[V_3W_3O_{19}]$[1] 2
      1.2.2 Synthesis of Anderson-Evans-Type $Na_9[TeV_3W_3O_{24}]$[3] 3
      1.2.3 Synthesis of Keggin-Type $Na_6[PV_3W_9O_{40}]$[4] 3
      1.2.4 Synthesis of Wells-Dawson-Type $Na_9[P_2V_3W_{15}O_{62}]$[6] 4
   1.3 Membrane setup 6
2. Results & discussion 8
   2.1 Membrane screening 8
   2.2 Stability of different POM structures 9
   2.3 Influence of parameters 15
      2.3.1 Concentration 15
      2.3.2 Membrane stability 17
      2.3.3 Flow rate and pressure 19
Literature 20

This supporting information contains 24 figures and 7 table on 21 pages.

1. Material and Methods

1.1 Catalyst characterization methods

During the POM catalyst synthesis, a Schott Instruments Lab 850 pH meter was used in combination with a Lab 850 with a BlueLine pH electrode from SI Analytics. For the membrane experiments, an EC-25 meter from Phoenix Instruments was used together with a gel pH plastic body electrode from XS Instruments.

Thermogravimetric analysis samples were submitted to the Fraunhofer Center for Applied Nanotechnology (CAN), Department of Physical Chemistry, at the University of Hamburg. Measurements were conducted using the NETZSCH TG 209 F1 220-10-039-K instrument and analyzed with NETZSCH PROTEUS software. Approximately 10 mg of the respective material was weighed into an Al_2O_3 crucible, and the mass change during heating was monitored. The sample was heated from 25 °C to 350 °C at a rate of 10 K/min. Prior to heating, the sample was tempered at 30 °C for 15 minutes and was held at 350 °C for 30 minutes after heating.

In addition, the incorporation of vanadium and phosphorus into the POM structures was determined using ^{51}V and ^{31}P-NMR. The ^{51}V- as well as ^{31}P-NMR measurements were conducted using a BRUKER AVANCE III HD 600 MHz spectrometer. The spectra obtained were analyzed using MESTERENOVA 10 software. ^{31}P NMR was performed with the following parameters: 512 scans and d1: 1 second ^{51}V NMR was conducted with the following parameters: 1024 scans, and d1: 0.3 seconds. For sample preparation 0.5 mL of catalyst solution was mixed with 0.1 D_2O.

1.2 Synthesis of catalysts

1.2.1 Synthesis of Lindqvist-Type $Na_5[V_3W_3O_{19}]$[1]

Sodium tungstate dihydrate (1.0 eq.) and vanadium pentoxide (1.0 eq.) were suspended demineralized water (100 mL) and heated to 80 °C. The pH was adjusted to dissolve the vanadium pentoxide from pH 7.9 to 12 with an aqueous NaOH solution. After stirring for 30 minutes the colorless reaction mixture was slowly adjusted to a pH value of 5.5 with 2 M hydrochloric acid. The reaction mixture was allowed to cool to room temperature and filtered. The desalting and purification of the crude product was carried out using membrane separation. Finally, the solvent was removed using a rotary evaporator and a yellow solid was obtained.

Characterization:

^{51}V-NMR (157.8 MHz, H_2O/D_2O, 25 °C, pH-value 5.9-6.5): δ [ppm] = -503, -509, - 516, -558, -571, -575. [2]

IR (ATR): $\tilde{\nu}$ [cm^{-1}] = 3250 (w, O-H, H_2O), 1630 (O-H, lattice H_2O), 934-935, 868 ($M{=}O_t$), 713 and 527 ($M{-}O_b{-}M$). [2]

Raman: $\tilde{\nu}$ [cm^{-1}] = 968, 955, 897, 817 ($M{=}O_t$), 327 (M-O-M), 265, 238 ($M{=}O_c$). [2]

ICP-OES: Calculated for $Na_5V_3W_3O_{19}$ · $8H_2O$: 12.06 % V, 43.51 % W. Found for $Na_5V_3W_3O_{19}$ · 7 H2O: 12.27 % V, 45.80 % W. The data are normalised by the expected molarity of tungsten. V/W ratio: 2.90/3.0.

TGA: 11 % weight loss upon drying, this corresponds to 7 mol water per mol of the POM.

1.2.2 Synthesis of Anderson-Evans-Type $Na_9[TeV_3W_3O_{24}]$[3]

The synthesis procedure was done according to Raabe et. al. Sodium metavanadate (3.01 eq.) was added and demineralized water (300 mL) was added. The white reaction mixture was heated at 60 °C for 20 minutes with stirring. A clear, colorless solution was obtained, to which sodium tungstate dihydrate (3.01 eq.) was added at a constant temperature. The solution remained clear and turned yellow. Telluric acid (1.0 eq.) was added to the solution immediately afterwards. The pH value was adjusted from the original 7.6 to 5.0 using 2 M hydrochloric acid (38 mL). During the addition, the reaction solution turned from yellow-orange to red. The entire addition was carried out at 70 °C. The resulting red solution was cooled to room temperature and the following day it was purified and desalted using membrane separation. Finally, the solvent was removed using a rotary evaporator and a light orange solid was obtained.

^{51}V-NMR (157.8 MHz, H_2O/D_2O, 25 °C, pH value 4.5-4.8): δ [ppm] = -503, -509, -516, -575.[3]

IR (ATR): $\tilde{\nu}$ [cm^{-1}] = 3350 (w, O-H, H_2O), 1621 (O-H, lattice H_2O), 949, 877 ($M{=}O_t$), 726, 681 (M-O_b-M), 639, 556 (Te-O). 483 (O-Te-O), 447 (M-O-M).[3]

Raman: $\tilde{\nu}$ [cm^{-1}] = 998, 963, 917 ($M{=}O_t$), 576, 415, 330 (M-O-M), 290, 262, 239, 217 ($M{=}O_c$). [3]

ICP-OES: Calculated for $Na_9TeV_3W_3O_{24}$ · $9H_2O$: 8.04 % Te, 9.63 % V, 34.82 % W. Found for $Na_9TeV_3W_3O_{24}$ · $9H_2O$: 4.21 % Te, 11.01 % V, 41.22 % W. The data are normalised by the expected molarity of tungsten. Te/V/W ratio: 0.8/2.8/3.

TGA: 10 % weight loss upon drying, this corresponds to 8 mol water per mol of the POM.

1.2.3 Synthesis of Keggin-Type $Na_6[PV_3W_9O_{40}]$[4]

Sodium tungstate dihydrate (6.1 eq.) was dissolved in demineralized water (400 mL) and an 85 % phosphoric acid solution (1.0 eq.) was added. The pH was 8.6. With stirring, glacial acetic acid (6.7 eq.) was added dropwise with stirring. During the addition, a colored precipitate formed. The pH value changed to 6.7 after complete addition.After two hours of stirring, the colorless precipitate was separated by vacuum filtration and stored dried in a desiccator for 24 hours. The intermediate product Na9xHx [PW_9O_{34}] · x H2O was obtained as a colorless solid. Sodium acetate (12.2 eq.) was dissolved in demineralized water (450 mL) and adjusted to a pH of 4.8 with acetic acid. Subsequently were added sodium metavanadate (3.2 eq.) and Na9 xHx [PW_9O_{34}] · x H2O (1.0 eq.) were added to the solution and stirred for 48 hours. The color of the solution changed to dark red during this time. It was filtered and then desalted by membrane filtration. In the final step, the solvent was removed using a ratory evaporator and a red powder was obtained.

^{31}P-NMR (242.9 MHz, H_2O/D_2O, 25 °C, pH-value 7.2-7.4): *δ* [ppm] = -12, -12.33, -12.79, -13.39, -13.65, -13.77. [5]

^{51}V-NMR (157.8 MHz, H_2O/D_2O, 25 °C, pH-value 7.2-7.4): *δ* [ppm] =-491, -497, -500. -502, -509, -525 (broad), -529, -543, -549, -551, -553, -556. [5]

IR (ATR): $\tilde{\nu}$ [cm^{-1}] = 3400 (w, O-H, H_2O), 1616 (O-H, lattice H_2O), 1068 (w, P-O), 947 ($M=O_t$), 858 ($M-O_b-M$), 746 ($M-O_c-M$). [5]

Raman: $\tilde{\nu}$ [cm^{-1}] = 997, 890 ($M=O_t$), 791; 259, 235 ($M=O_c$).

ICP-OES: Calculated for $Na_6PV_3W_9O_{40} \cdot 15\ H_2O$: 1.18 % P, 5.84 % V, 63.23 % W. Found for $Na_6PV_3W_9O_{40} \cdot 16H_2O$: 1.29 % P, 6.06 % V, 55.59 % W. The data are normalised by the expected molarity of tungsten. P/V/W ratio: 1.23/3.52/9.

TGA: 10 % weight loss upon drying, this corresponds to 15 mol water per mol of the POM.

1.2.4 Synthesis of Wells-Dawson-Type $Na_9[P_2V_3W_{15}O_{62}]$[6]

Sodium tungstate dihydrate (0.9 eq.) was acidified in demineralised water (350 mL) and by fractional addition of 4 M hydrochloric acid (1.0 eq.) with vigorous stirring. When the cloudy solution cleared, 4 M phosphoric acid (0.9 eq.) was slowly added. The light yellow, clear solution was heated under reflux for at least 24 hours. After this reaction time in the yellow colour of the solution intensified. The reaction solution was allowed to cool to room temperature and potassium chloride (150 g) was added. The resulting precipitate was removed by vacuum filtration and dried. The resulting crude product was dissolved in demineralised water (650 mL) and insoluble impurities were filtered off. The clear solution was heated to approximately 80 °C for at least 72 hours. The reaction solution was then cooled to room temperature before being stored at 4 °C. After this time, yellow crystals of the α-$K_6P_2W_7O_{62} \cdot 14\ H_2O$ structure were obtained. The synthesised α-$K_6P_2W_7O_{62} \cdot 14\ H_2O$ structure (1.0 eq.) was dissolved in demineralised water (150 mL) and sodium perchlorate monohydrate (18.3 eq.) was added. After 20 minutes of vigorous stirring, the mixture was cooled in an ice bath. The resulting potassium perchlorate was removed after three hours by filtration. A solution of sodium perchlorate dissolved in demineralised water (100 mL) sodium carbonate (7.34 eq.) was added to the filtrate. A white precipitate was formed, which was decanted, filtered by means of a medium-porous, sintered glass frit and then dried for about three hours using vacuum filtration. The precipitate was then rinsed for one to two minutes with a solution of sodium chloride in demineralised water (30 mL) for one to two minutes and dried by suction for a further three hours. Afterwards the precipitate was washed with ethanol (30 mL) for two to three minutes and dried again by air-dried again by vacuum filtration for three hours. Purification using ethanol and subsequent drying was carried out twice. After three days of drying in air, the intermediate product α-$Na_{12}P_2W_{15}O_{56} \cdot 24\ H_2O$ was obtained. The intermediate α-$Na_{12}P_2W_{15}O_{56} \cdot 24\ H_2O$ (1.0 eq.) was dissolved in demineralised water (120 mL) at 100 °C. Simultaneously, sodium metavanadate (3.3 eq.) was dissolved separately at 100 °C in demineralised water (120 mL). Both solutions were combined and the pH value was adjusted by

adding 4 M hydrochloric acid.The solution turned orange in colour. The crude product was worked up with the membrane system as described in the previous synthesis.

^{31}P-NMR (242.9 MHz, H_2O/D_2O, 25 °C, pH-value 6.9-7.3): δ [ppm] = -11.46, -12.37, -13.06, -13.46, -13.56, -14.17, -14.34, -14.44. [7]

^{51}V-NMR (157.8 MHz, H_2O/D_2O, 25 °C, pH-value 6.9-7.3): δ [ppm] = -526, -540, -547, -553, -554, -556, -558.

IR (ATR): $\tilde{\nu}$ [cm^{-1}] = 3400 (w, O-H, H_2O), 1616 (O-H, lattice H_2O), 1081,1054 (P-O_a), 952 (M=O_d), 882 (M-O_b-M), 726 (M-O_c-M).

Raman: $\tilde{\nu}$ [cm^{-1}] = 1020, 997, 970 ,904 (M=O_t), 375, 309 (M-O-M), 266, 236 (M=O_c). [7]

ICP-OES: Calculated for $Na_9P_2V_3W_{15}O_{62} \cdot 20H_2O$: 1.49 % P, 3.68 % V, 66.55 % W. Found for $Na_9P_2V_3W_{15}O_{62} \cdot 19\ H_2O$: 1.36 % P, 3.74 % V, 60.63 % W The data are normalized by the expected molarity of tungsten. P/V/W ratio: 1.92/3.76/15.

TGA: 8 % weight loss upon drying, this corresponds to 19 mol water per mol of the POM.

1.3 Membrane setup

The flow diagram and pictures of the used membrane setup can be seen below. By closing valve V107 pressure can be applied via the adjusted by the pressure relief valve V106. Another option to apply pressure is by turning valve V102 and adjusting fine dosing valve V103. The Valve V105 can be used either to recycle the permeate stream back into the feed or collect the permeate for measurements. The same functionality applies to valve V104 for the retentate stream. The membrane module has an integrated stirrer and is stirred during the process. The active area of the membrane is 33 cm^2.

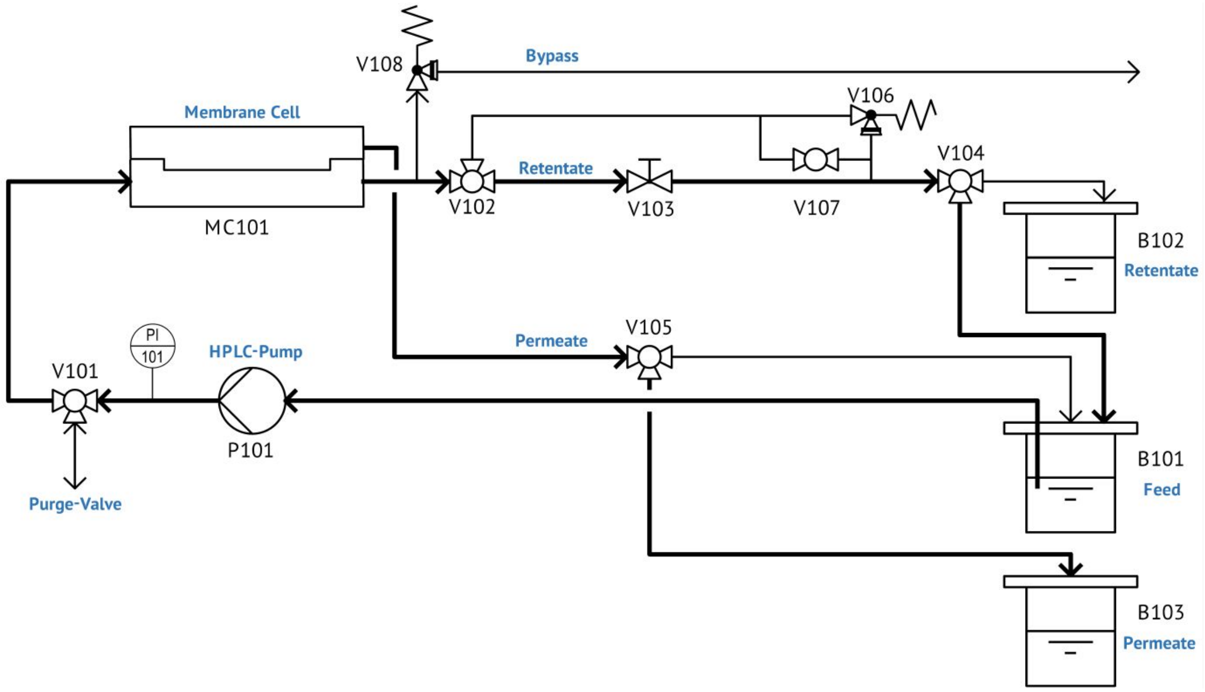

Figure S1: Flow diagram of used membrane setup.

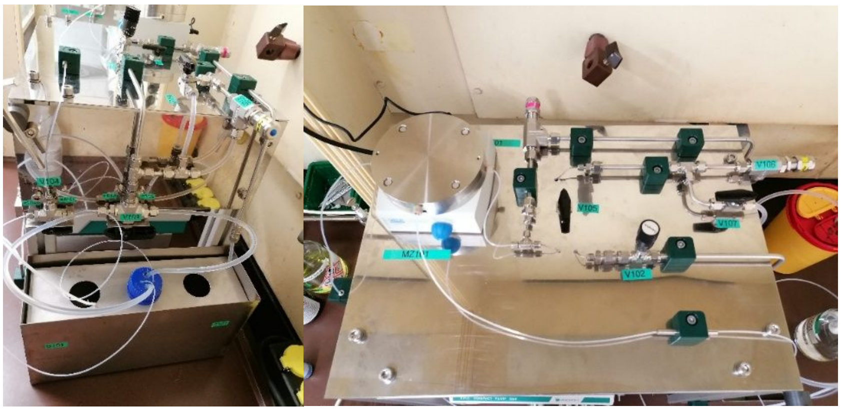

Figure S2: Pictures of the used membrane setup. Side view (left), view from above (right).

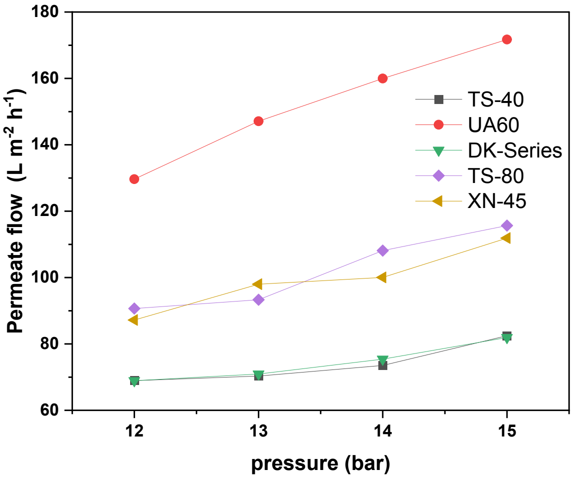

Figure S3 Pure water permeability of used membranes. *Experimental conditions*: pre-wetted membranes, ambient temperature 15 mL min^{-1} pump flow, 1100 rpm.

Table S1: Overview of used membranes for the nanofiltration experiments.

**Membrane**	**Cut-Off (Da)**	**pH-range**	**$MgSO_4$ rejection (%)**	**NaCl rejection (%)**	**Isoelectric point**	**Zeta potential (mV) pH 7**
TS-80	150-200[8]	1.0-12.0[8]	98.5[8]	80[8]	2.5[14]	-14[14]
DK-Series	150-300[9]	2.0-10.0[9]	98.0[9]	-*	4.5[13]	-7[13]
TS-40	200-300[10]	1.0-12.0[10]	98.5[10]	40[10]	2.5[14]	-40 (pH=8)[14]
XN-45	300-500[11]	1.0-12.0[11]	96[11]	20[11]	4.5[13]	-27[13]
UA60	1000[12]	1.0-12.0[12]	80[12]	-*	-*	-*

* Data not published from supplier or found in literature.

2. Results & discussion

2.1 Membrane screening

Table S2: Rejection of the Keggin type metals and permeate flow rate for different membranes.

**Membrane**	**Cut-Off (Da)**	**Tungsten (%)**	**Vanadium (%)**	**Phosphorus (%)**	**Permeate flow ($L\ m^{-2}\ h^{-1}$)**
TS-80	150-200	99	99	99	191
DK-Series	150-300	99	99	99	237
TS-40	200-300	99	99	99	188
XN-45	300-500	99	99	99	202
UA 60	1000	99	99	97	209

Experimental conditions: pre-wetted membranes, ambient temperature,
5 mM $Na_6[PV_3W_9O_{40}]$, 100 mL H_2O, 15 mL · min^{-1} pump flow, p = 32 bar, 1100 rpm.

Table S3: Rejection of the Wells-Dawson type metals and permeate flow rate for different membranes.

**Membrane**	**Cut-Off (Da)**	**Tungsten (%)**	**Vanadium (%)**	**Phosphorus (%)**	**Permeate flow ($L\ m^{-2}\ h^{-1}$)**
TS-80	150-200	99	99	99	176
DK-Series	150-300	99	99	99	237
TS-40	200-300	99	99	99	149
XN-45	300-500	99	99	99	202
UA60	1000	99	99	97	210

Experimental conditions: pre-wetted membranes, ambient temperature,
5 mM $Na_9[P_2V_3W_{15}O_{62}]$, 100 mL H_2O, 15 mL · min^{-1} pump flow, p = 32 bar, 1100 rpm.

2.2 Stability of different POM structures

2.2.1 Lindqvist

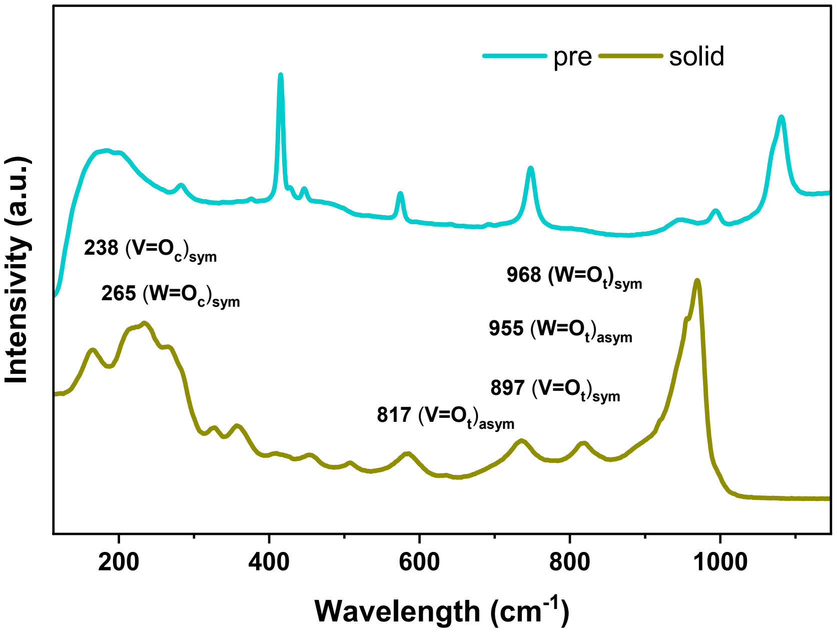

Figure S4: Raman spectra of solid $Na_5[V_3W_3O_{19}]$ vs raman spectra of dissolved $Na_5[V_3W_3O_{19}]$ in H2O.

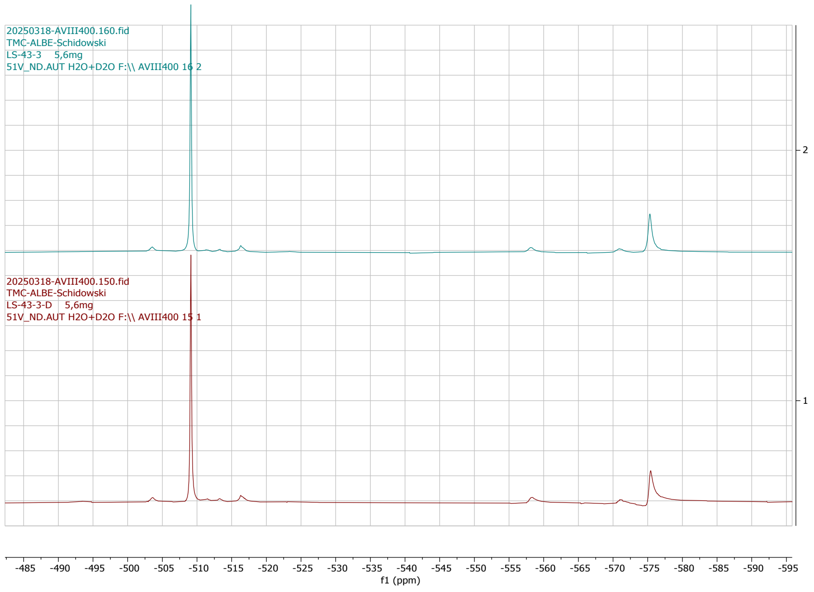

Figure S5: ^{51}V NMR spectra before (top) and after (below) the membrane separation of $Na_5[V_3W_3O_{19}]$.

2.2.2 Anderson-Evans

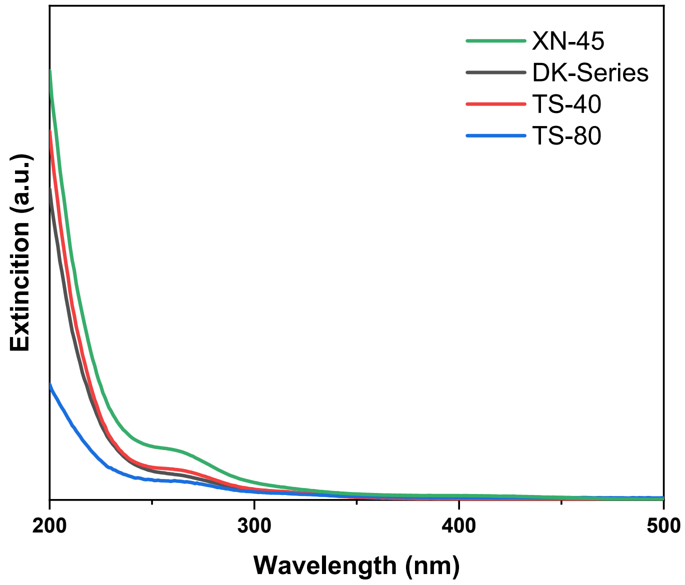

Figure S6: UV/Vis spectra of undiluted permeate solution after the membrane separation of $Na_9[TeV_3W_3O_{24}]$ for DK-Series (black), TS-40 (red), TS-80 (blue) and XN-45 (green).

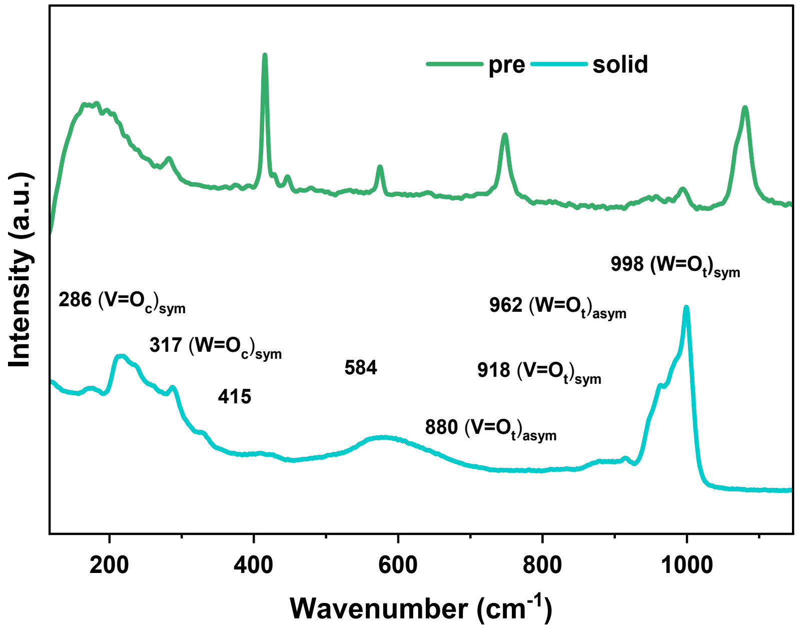

Figure S7*:* Raman spectra of solid $Na_7[TeV_3W_3O_{24}]$ vs raman spectra of dissolved $Na_7[TeV_3W_3O_{24}]$ in H_2O.

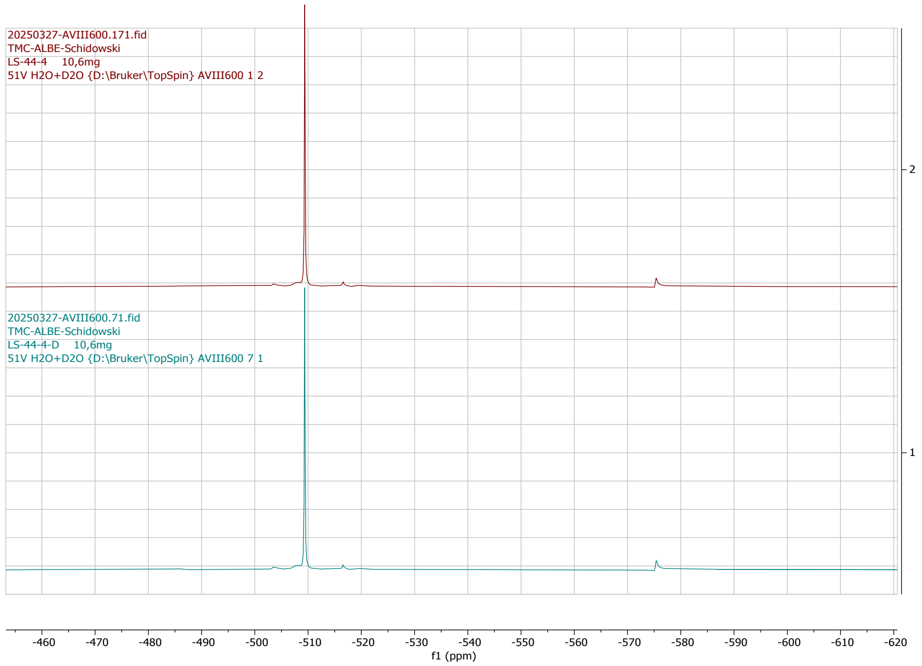

Figure S8: ^{51}V NMR spectra before (top) and after (below) the membrane separation of $Na_7[TeV_3W_3O_{24}]$.

2.2.3 Keggin

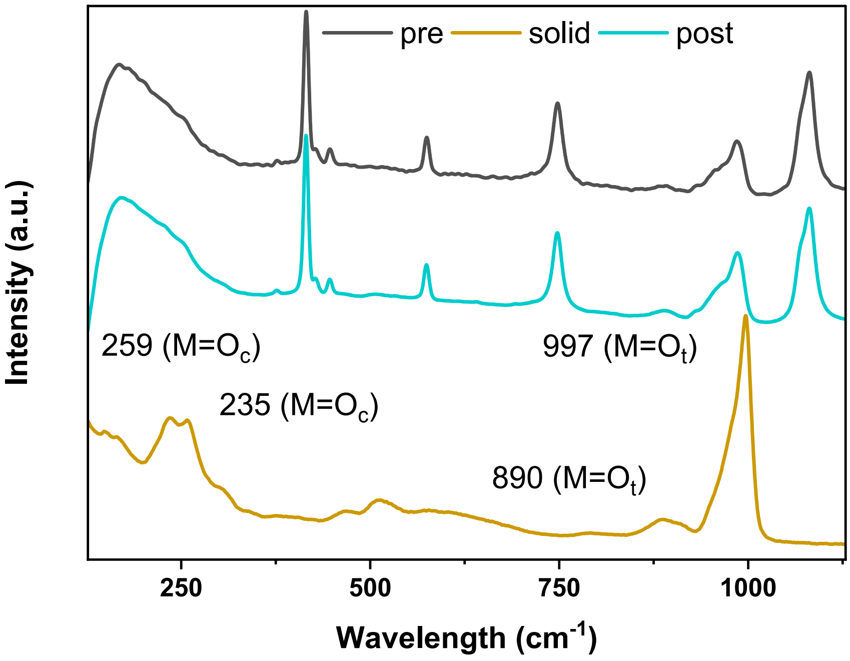

Figure S9: Raman spectra of solid $Na_6[PV_3W_9O_{40}]$ vs raman spectra dissolved $Na_6[PV_3W_9O_{40}]$ in H_2O.

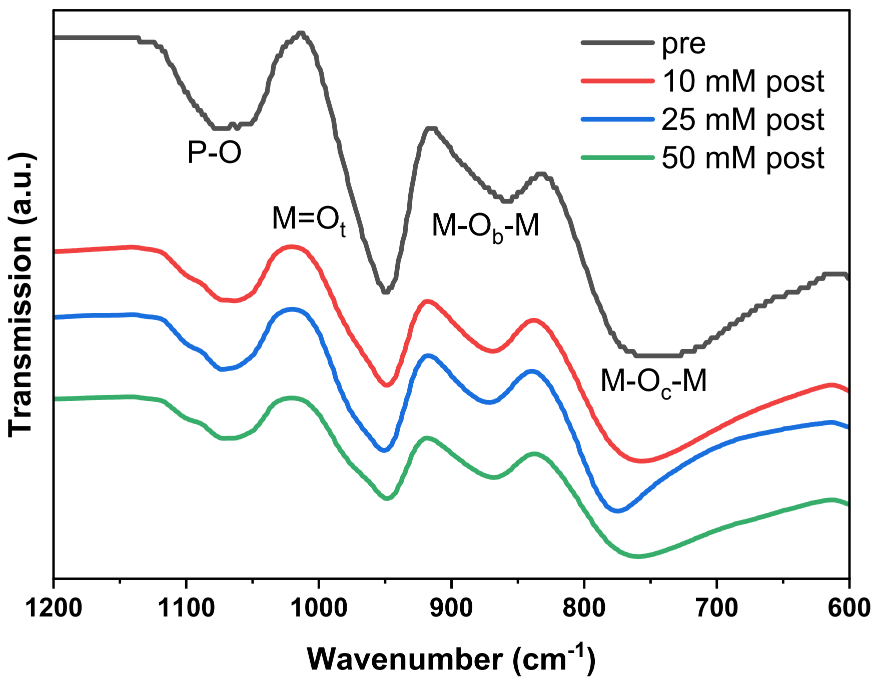

Figure S10*:* FT-IR of solid $Na_6[PV_3W_9O_{40}]$.

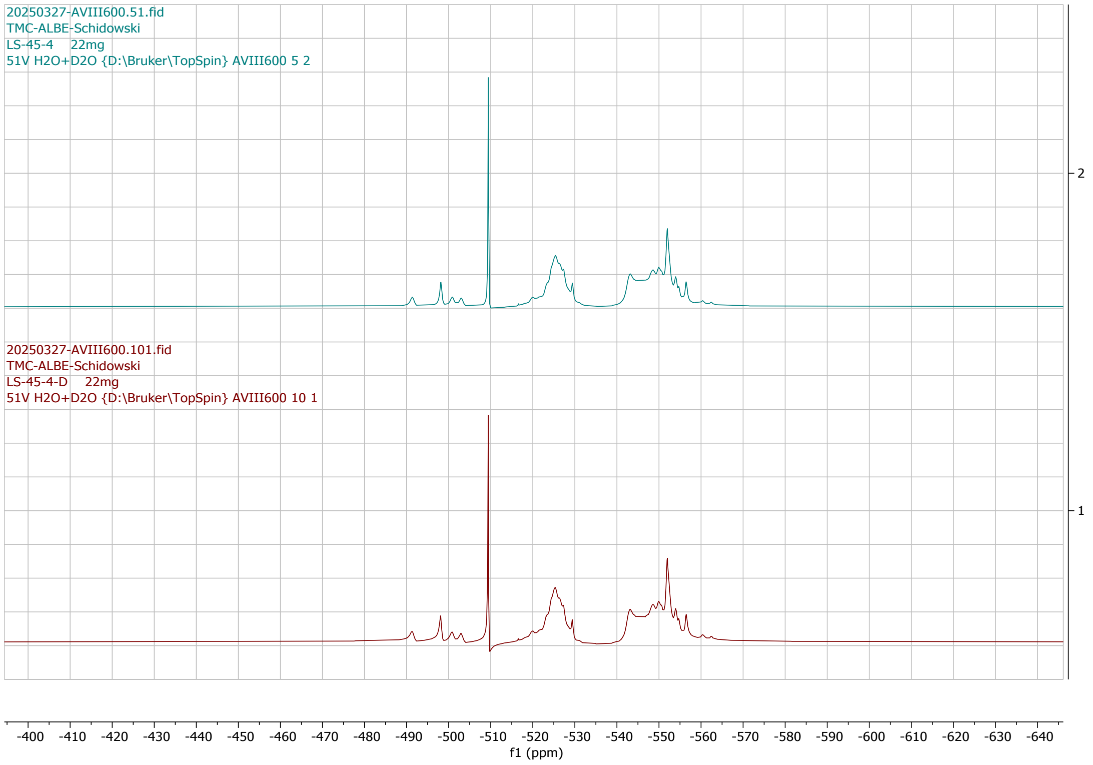

Figure S11*:* ^{51}V NMR spectra before (top) and after (below) the membrane separation of $Na_6[PV_3W_9O_{40}]$.

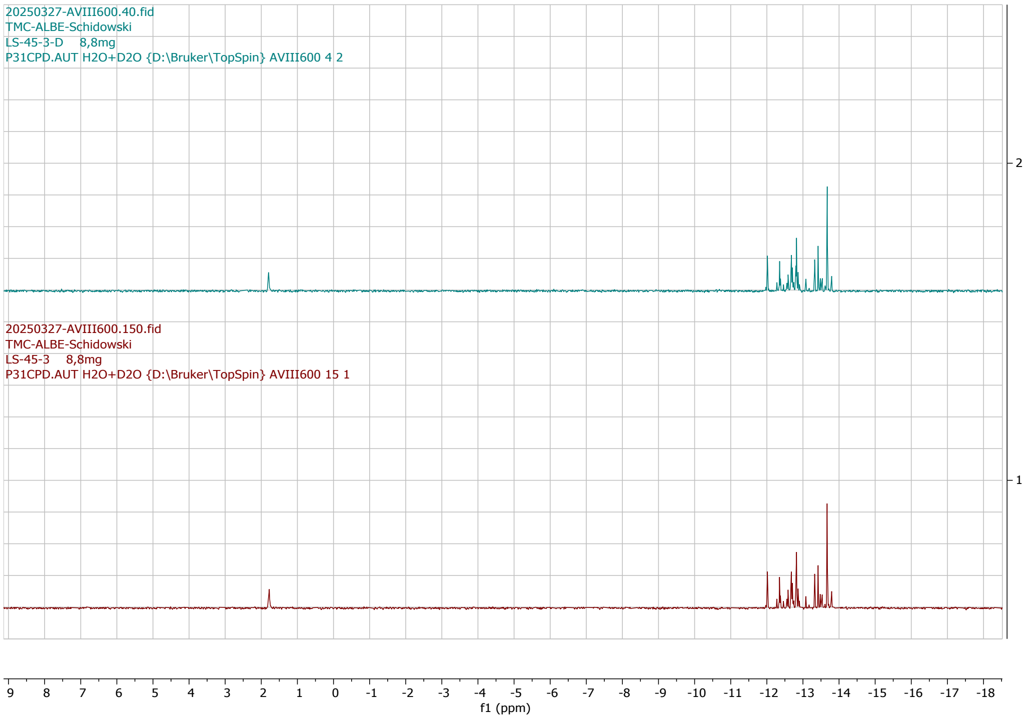

Figure S12*:* ^{31}P NMR spectra before (top) and after (below) the membrane separation of $Na_6[PV_3W_9O_{40}]$.

2.2.4 Wells-Dawson

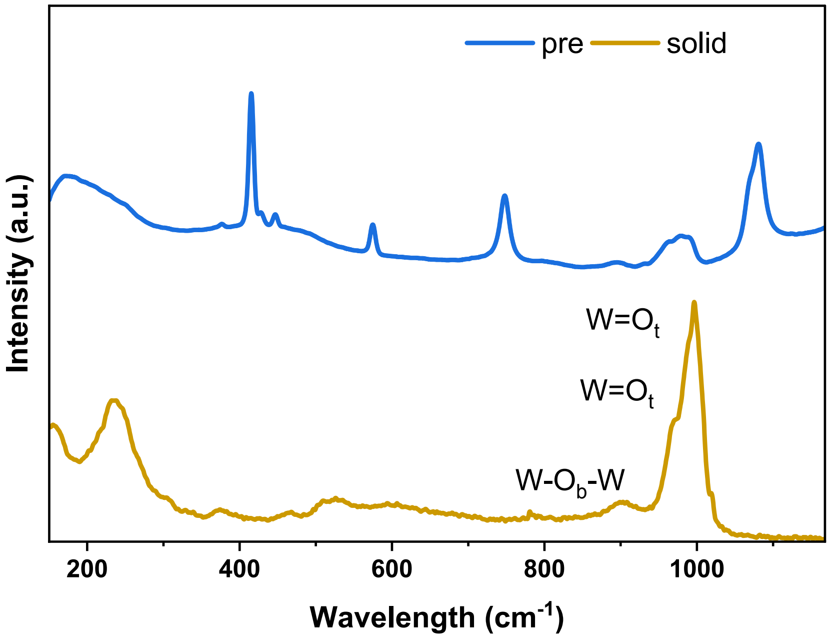

Figure S13: Raman spectra of solid $Na_9[P_2V_3W_{15}O_{62}]$ vs raman spectra of dissolved $Na_9[P_2V_3W_{15}O_{62}]$ in H_2O.

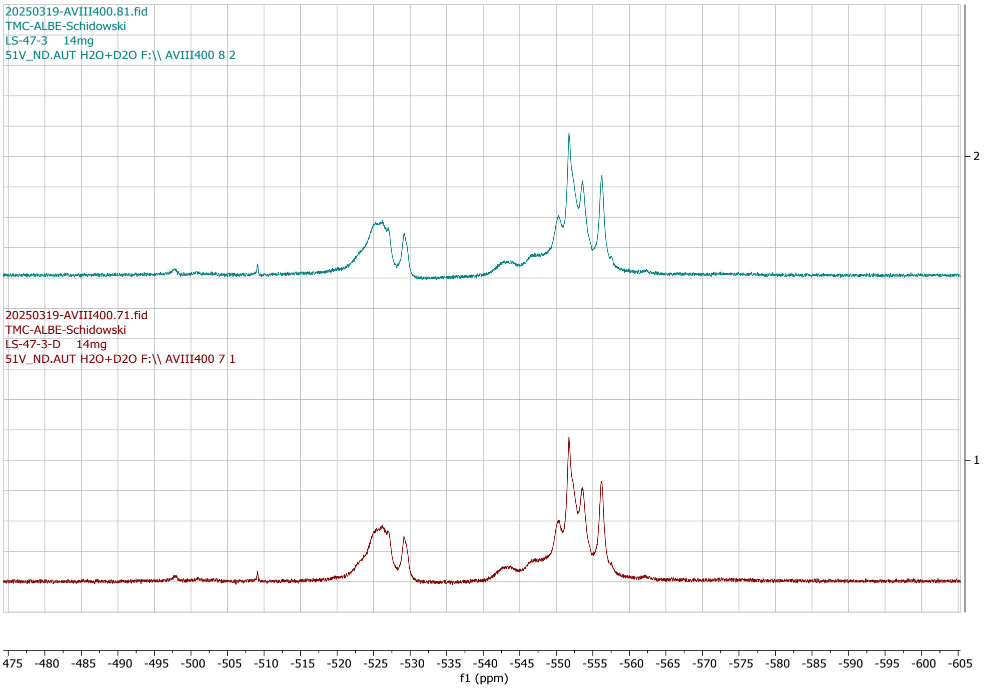

Figure S14: ^{51}V NMR spectra before (top) and after (below) the membrane separation of $Na_9[P_2V_3W_{15}O_{62}]$.

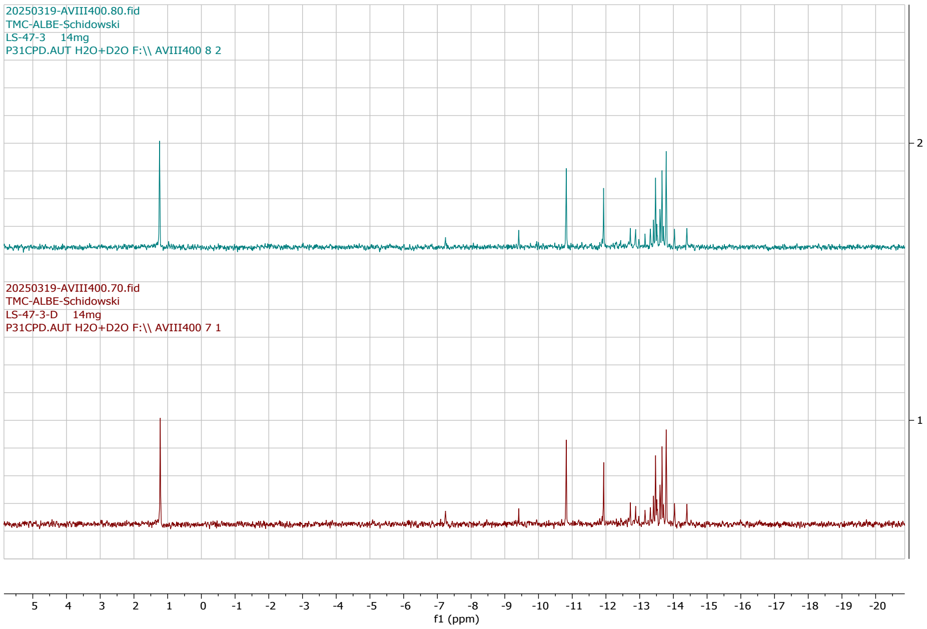

Figure S15: ^{31}P NMR spectra before (top) and after (below) the membrane separation of $Na_9[P_2V_3W_{15}O_{62}]$.

2.3 Influence of parameters

2.3.1 Concentration

Table S4: Rejection of the Lindqvist type metals and permeate flow for different feed concentration with TS-80 membrane.

**Feed concentration (mM)**	**Tungsten (%)**	**Vanadium (%)**	**Permeate flow (L m^{-2} h^{-1})**	**Permeate compostion**
1	98.0	98.9	245	38/5/3
5	99.5	99.6	225	11/3/3
10	99.5	99.7	198	7/3/3
25	99.4	99.5	176	4/3/3
50	99.6	99.7	149	3/3/3

Experimental conditions: pre-wetted TS-80 membrane, ambient temperature, catalyst $Na_5[V_3W_3O_{19}]$, 100 mL H_2O, 15 mL · min^{-1} pump flow, p = 32 bar, 1100 rpm.

Table S5: Rejection of the Keggin type metals and permeate flow for different feed concentration with TS-80 membrane.

**Feed concentration (mM)**	**Tungsten (%)**	**Vanadium (%)**	**Phoshporus (%)**	**Permeate flow (L m^{-2} h^{-1})**	**Permeate compostion**
1	99.4	97.4	96.8	227	6/16/9
5	99.9	99.6	99.5	232	6/16/9
10	99.8	99.7	99.7	198	1/3.5/9
25	99.8	99.8	99.8	163	1/3.5/9
50	99.8	99.8	99.8	120	1/3.5/9

Experimental conditions: pre-wetted TS-80 membrane, ambient temperature, catalyst $Na_5[V_3W_3O_{19}]$, 100 mL H_2O, 15 mL · min^{-1} pump flow, p = 32 bar, 1100 rpm.

Table S6: Rejection of the Wells-Dawson type metals and permeate flow for different feed concentration with TS-80 membrane.

**Feed concentration (mM)**	**Tungsten (%)**	**Vanadium (%)**	**Phoshporus (%)**	**Permeate flow (L m^{-2} h^{-1})**	**Permeate compostion**
1	99.9	99.7	99.8	230	4/11/15
5	99.8	99.9	99.9	221	2/3/15
10	99.8	99.9	99.8	165	2/3/15
25	99.8	99.8	99.8	135	2/3/15
50	99.8	99.8	99.8	91	2/3/15

Experimental conditions: pre-wetted TS-80 membrane, ambient temperature, catalyst $Na_5[V_3W_3O_{19}]$, 100 mL H_2O, 15 mL · min^{-1} pump flow, p = 32 bar, 1100 rpm.

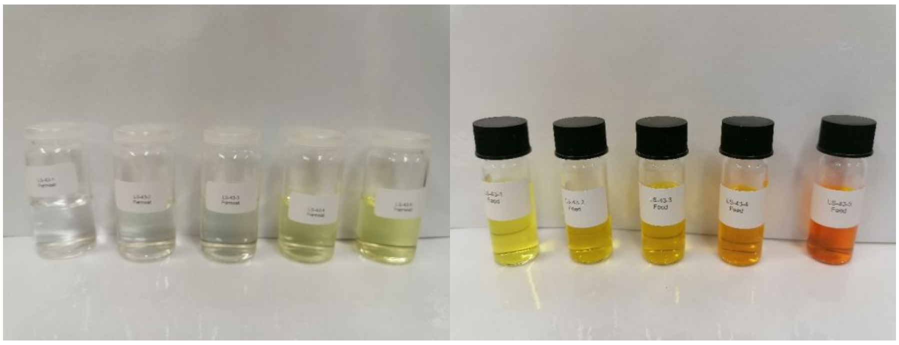

Figure S16: Permeate solution (left) and feed solution (right) of $Na_5[V_3W_3O_{19}]$ for 1 mM to 50 mM.

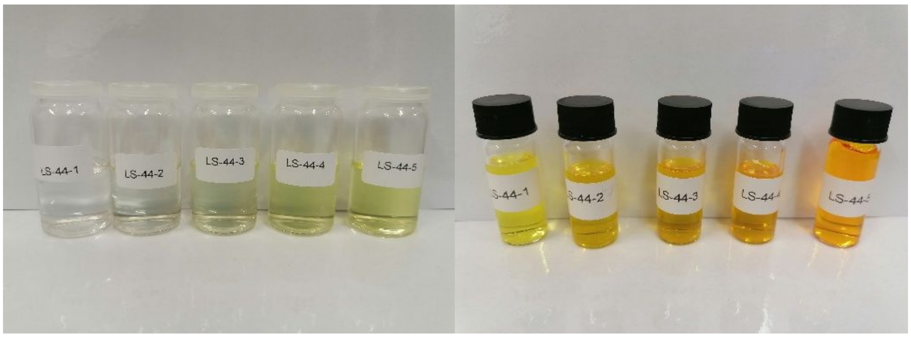

Figure S17: Permeate solution (left) and feed solution (right) of $Na_9[TeV_3W_3O_{24}]$ for 1 mM to 50 mM.

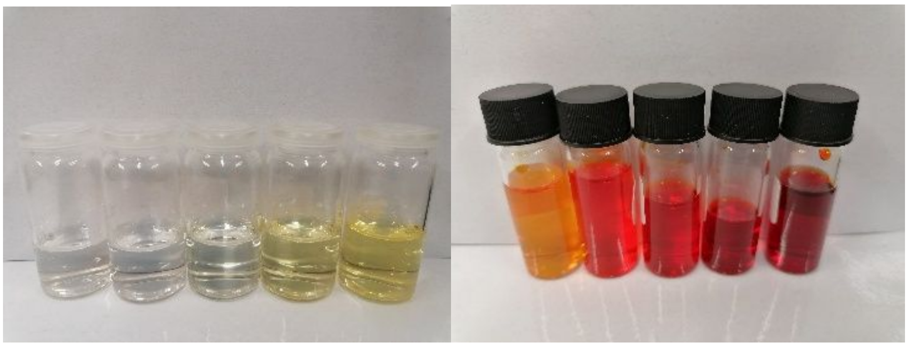

Figure S18: Permeate solution (left) and feed solution (right) of $Na_6[PV_3W_9O_{40}]$ for 1 mM to 50 mM.

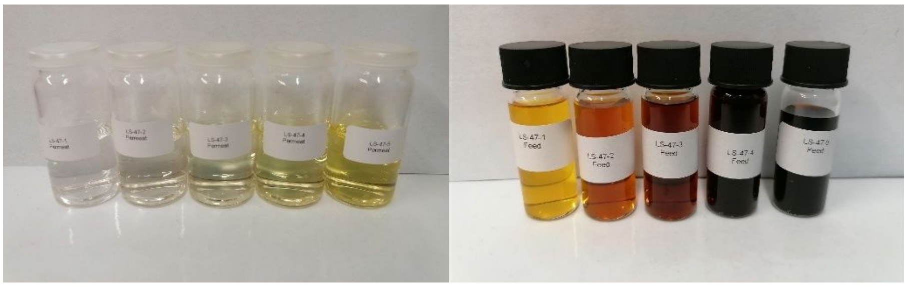

Figure S19: Permeate solution (left) and feed solution (right) of $Na_9[P_2V_3W_{16}O_{62}]$ for 1 mM to 50 mM.

2.3.2 Membrane stability

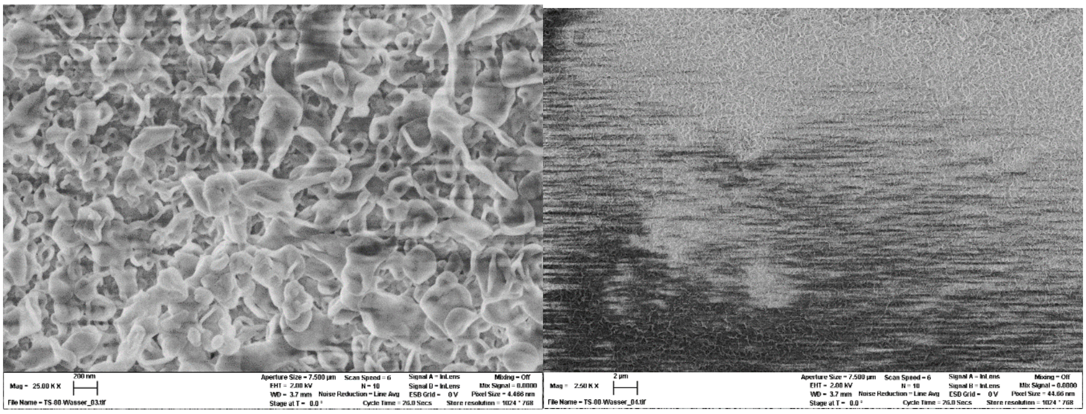

Figure S20: SEM measurement of fresh TS-80 membrane.

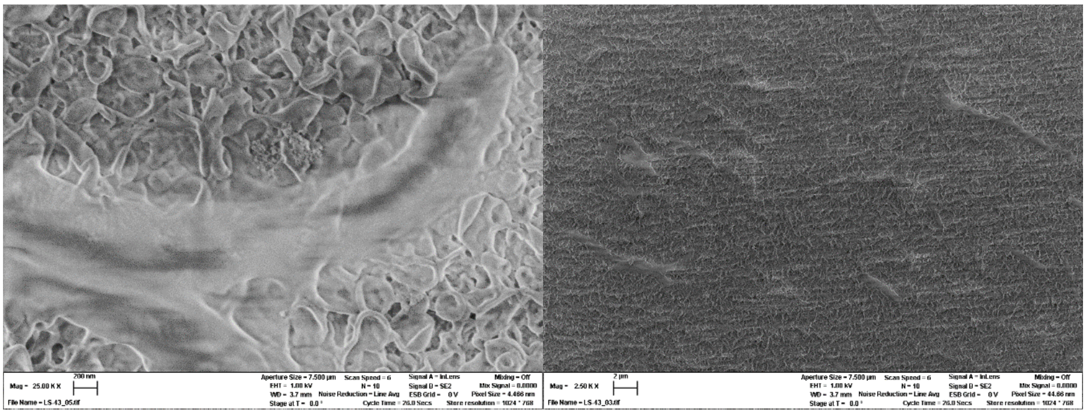

Figure S21: SEM measurements of used membrane after testing the recovery of $Na_5[V_3W_3O_{19}]$ with a feed concentration of 50 mM. The membrane was flushed with water and dried before measurement.

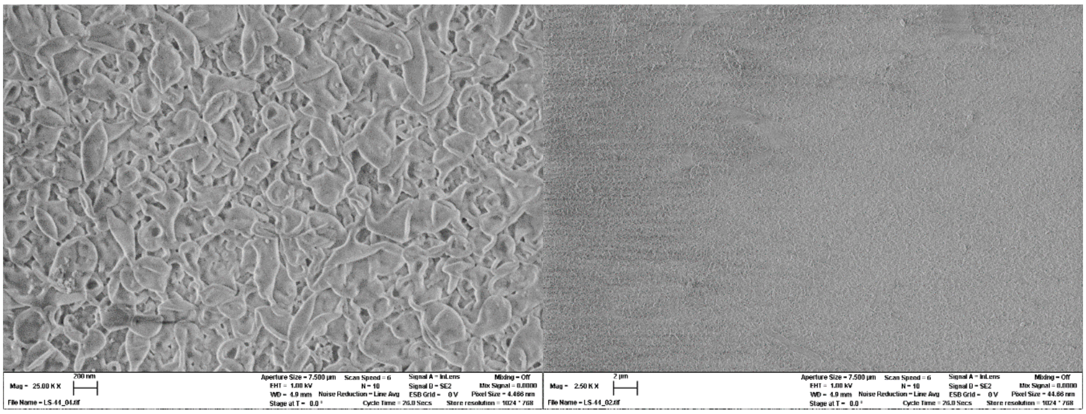

Figure S22: SEM measurements of used membrane after testing the recovery of $Na_9[TeV_3W_3O_{19}]$ with a feed concentration of 50 mM. The membrane was flushed with water and dried before measurement.

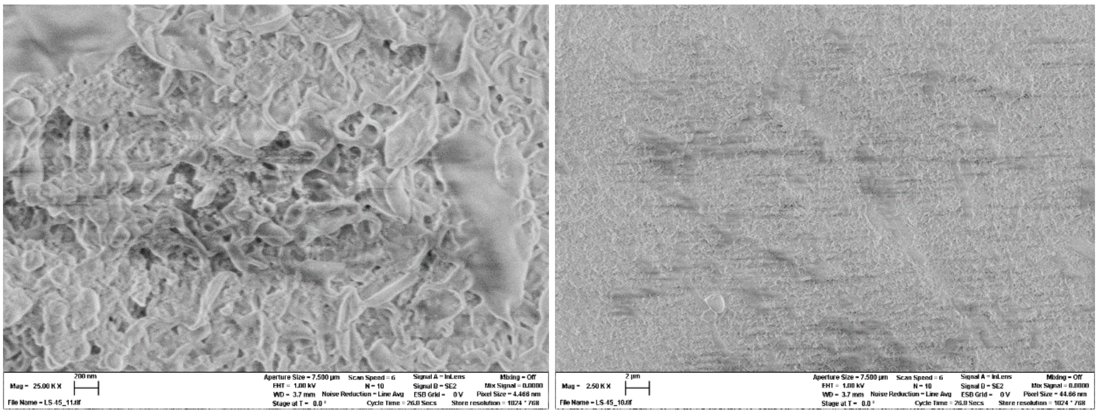

Figure S23: SEM measurements of used membrane after testing the recovery of $Na_6[PV_3W_9O_{40}]$ with a feed concentration of 50 mM. The membrane was flushed with water and dried before measurement.

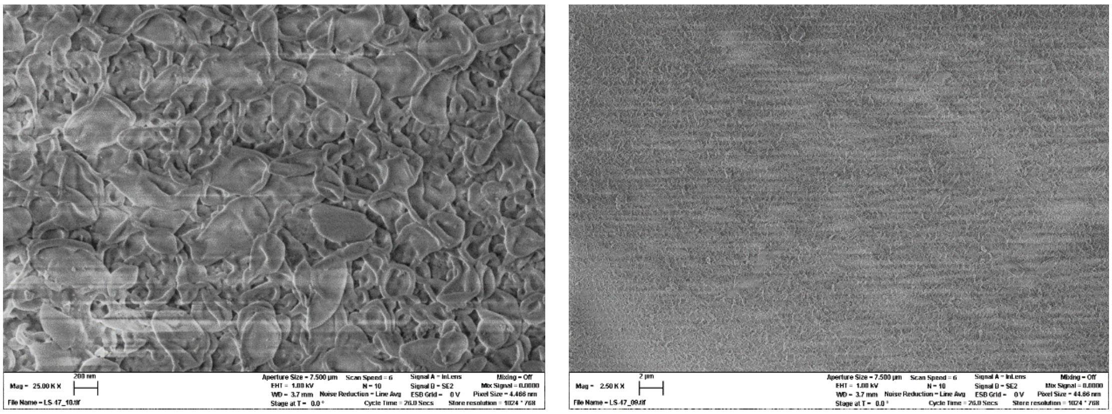

Figure S24: SEM measurements of used membrane after testing the recovery of $Na_9[P_2V_3W_{16}O_{62}]$ with a feed concentration of 50 mM. The membrane was flushed with water and dried before measurement.

2.3.3 Flow rate and pressure

Table S7: Rejection of the Anderson-Evans Type metals and permeate flow under different process conditions.

**Pressure (bar)**	**Flow rate (mL · min^{-1})**	**Tellurium (%)**	**Tungsten (%)**	**Vanadium (%)**	**Permeate flow (L m^{-2} h^{-1})**
32	10	97.3	99	99	182
32	12.5	97.2	99	99	179
32	15	98.2	99	99	185
32	17.5	98.3	99	99	195
32	20	98.1	99	99	191
15	15	99.2	99	99	103
20	15	99.1	99	99	133
25	15	98.9	99	99	163
30	15	98.7	99	99	190
35	15	98.6	99	99	195

Experimental conditions: pre-wetted TS-80 membrane, ambient temperature, 5 mM catalyst $Na_9[TeV_3W_3O_{24}]$, 100 mL H_2O, 1100 rpm.

Table S8: Determination of the standard deviation for the rejection of the Anderson-Evans Type metals and permeate flow under optimist process conditions.

Run	Tellurium (%)	Tungsten (%)	Vanadium (%)	Permeate flow ($L\ m^{-2}\ h^{-1}$)
1	99.1	> 99	> 99	260
2	98.7	> 99	> 99	273
3	98.9	> 99	> 99	265

Experimental conditions: pre-wetted TS-80 membrane, ambient temperature, catalyst $Na_9[TeV_3W_3O_{24}]$, 100 mL H_2O, 20 mL · min^{-1} pump flow, p = 35 bar, 1100 rpm.

The standard deviation tellurium rejection is ± 0.2 % and for the permeate flow is ± 4 L · m^{-2} · h^{-1}.

Literature

(1) Albert, J.; Mehler, J.; Tucher, J.; Kastner, K.; Streb, C. One–Step Synthesizable Lindqvist−isopolyoxometalates as Promising New Catalysts for Selective Conversion of Glucose as a Model Substrate for Lignocellulosic Biomass to Formic Acid. *ChemistrySelect* **2016**, *1* (11), 2889–2894. https://doi.org/10.1002/slct.201600797.

(2) Yerra, S.; Amanchi, S. R.; Das, S. K. Synthesis and Structural Characterization of Lindqvist Type Mixed-Metal Cluster Anion [V2W4O19] 4− in Discrete and Coordination Polymer Compounds. *J Mol Struct* **2014**, *1062*, 53–60. https://doi.org/10.1016/j.molstruc.2014.01.005.

(3) Raabe, J. C.; Esser, T.; Poller, M. J.; Albert, J. Synthesis and Characterization of V Substituted Anderson-Type Telluro-Molybdates and Tungstates for Catalytic Oxidation of Furan Derivatives to Formic and Maleic Acid. *Catalysis Today*. Elsevier B.V. November 1, 2024. https://doi.org/10.1016/j.cattod.2024.114899.

(4) Domaille, P. J.; Watunya, G. Synthesis and Tungsten-183 NMR Characterization of Vanadium-Substituted Polyoxometalates Based on B-Type Tungstophosphate PW9O349-Precursors. *Inorg Chem* **1986**, *25* (8), 1239–1242. https://doi.org/10.1021/ic00228a033.

(5) Raabe, J. C.; Aceituno Cruz, J.; Albert, J.; Poller, M. J. Comparative Spectroscopic and Electrochemical Study of V(V)-Substituted Keggin-Type Phosphomolybdates and -Tungstates. *Inorganics (Basel)* **2023**, *11* (4). https://doi.org/10.3390/inorganics11040138.

(6) Mbomekallé, I.; Lu, Y.-W.; Keita, B.; Nadjo, L. Simple, High Yield and Reagent-Saving Synthesis of Pure α-K6P2W18O62 · 14H2O. *Inorg Chem Commun* **2004**, *7*, 86–90. https://doi.org/10.1016/j.inoche.2003.10.011.

(7) Ueda, T.; Nishimoto, Y.; Saito, R.; Ohnishi, M.; Nambu, J. Vanadium (V)-Substitution Reactions of Wells–Dawson-Type Polyoxometalates: From [X2M18O62] 6−(X= P, As; M= Mo, W) to [X2VM17O62] 7−. *Inorganics (Basel)* **2015**, *3* (3), 355–369. https://doi.org/10.3390/inorganics3030355.

(8) MANN+HUMMEL Water & Fluid Solutions GmbH. PRODUCT SPECIFICATION-TRISEP®TS-80 - Manufacturer Data Sheet. **2021**.

(9) SUEZ SA. *PRODUCT SPECIFICATION - DK Series Manufacturer Data Sheet*. www.lenntech.comFax.

(10) MANN+HUMMEL Water & Fluid Solutions GmbH. *PRODUCT SPECIFICATION-TRISEP®TS-40 - Manufacturer Data Sheet*; 2021. https://water-membrane-solutions.mann-hummel.com/content/dam/lse-wfs/product-related-assets/data-sheets/TS40.pdf (accessed 2025-07-29).

(11) MANN+HUMMEL Water & Fluid Solutions GmbH. PRODUCT SPECIFICATION-TRISEP® XN-45 - Manufacturer Data Sheet. **2021**.

(12) MANN+HUMMEL Water & Fluid Solutions GmbH. *PRODUCT SPECIFICATION-TRISEP® UA60 - Manufacturer Data Sheet*; **2021**. https://water-membrane-solutions.mann-hummel.com/content/dam/lse-wfs/product-related-assets/data-sheets/UA60.pdf (accessed 2025-07-29).

(13) Mandale, S.; Jones, M. Interaction of electrolytes and non-electrolytes in nanofiltration. *Desalination* **2008**, *219*, 262–271. https://doi.org/10.1016/j.desal.2007.06.005.

(14) Żyłła, R.; Foszpańczyk, M.; Kamińska, I.; Kudzin, M., Balcerzak; J., & Ledakowicz, S. Impact of Polymer Membrane Properties on the Removal of Pharmaceuticals. *Membranes*. **2022** *12*, 150. https://doi.org/10.3390/membranes12020150.
